# Phylogenetic relationship of prophages is affected by CRISPR selection in Group A *Streptococcus*

**DOI:** 10.1186/s12866-019-1393-y

**Published:** 2019-01-28

**Authors:** Shunsuke Yamada, Masaki Shibasaki, Kazunori Murase, Takayasu Watanabe, Chihiro Aikawa, Takashi Nozawa, Ichiro Nakagawa

**Affiliations:** 1 Department of Microbiology, Graduate School of Medicine, Kyoto University, Kyoto, 606-8501 Japan; 2 Department of Oral Implantology and Regenerative Dental Medicine, Graduate School of Medical and Dental Sciences, Tokyo Medical and Dental University, Tokyo, 113-8510 Japan; 3 Department of Infectious Diseases, Faculty of Medicine, Miyazaki University, Miyazaki, 889-1692 Japan; 4 Department of Chemistry, School of Dentistry, Nihon University, Tokyo, 101-8310 Japan

**Keywords:** CRISPR/*cas*, Prophage, Group a *Streptococcus*

## Abstract

**Background:**

Group A *Streptococcus* (GAS) is a major human pathogen, which is associated with a wide spectrum of invasive diseases, such as pharyngitis, scarlet fever, rheumatic fever, and streptococcal toxic shock syndrome (STSS). It is hypothesized that differences in GAS pathogenicity are related to the acquisition of diverse bacteriophages (phages). Nevertheless, the GAS genome also harbors clustered regularly interspaced short palindromic repeats (CRISPR) and CRISPR-associated (*cas*) genes, which play an important role in eliminating foreign DNA, including those of phages. However, the structure of prophages in GAS strains is mosaic, and the phylogenetic relationship between prophages and CRISPR is not clear. In this study, we analyzed CRISPR and prophage structure using 118 complete genome sequences of GAS strains to elucidate the relationship between two genomic elements. Additionally, phylogenetic and M-type analyses were performed.

**Results:**

Of the 118 GAS strains, 80 harbored type I-C and/or II-A CRISPR/*cas* loci. A total of 553 spacer sequences were identified from CRISPR/*cas* loci and sorted into 229 patterns. We identified and classified 373 prophages into 14 groups. Some prophage groups shared a common integration site, and were related to M-type. We further investigated the correlation between spacer sequences and prophages. Of the 229 spacer sequence patterns, 203 were similar to that of other GAS prophages. No spacer showed similarity with that of a specific prophage group with *mutL* integration site. Moreover, the average number of prophages in strains with type II-A CRISPR was significantly less than that in type I-C CRISPR and non-CRISPR strains. However, there was no statistical difference between the average number of prophages in type I-C strains and that in non-CRISPR strains.

**Conclusions:**

Our results indicated that type II-A CRISPR may play an important role in eliminating phages and that the prophage integration site may be an important criterion for the acceptance of foreign DNA by GAS. M type, spacer sequence, and prophage group data were correlated with the phylogenetic relationships of GAS. Therefore, we hypothesize that genetic characteristics and/or phylogenetic relationships of GAS may be estimated by analyzing its spacer sequences.

**Electronic supplementary material:**

The online version of this article (10.1186/s12866-019-1393-y) contains supplementary material, which is available to authorized users.

## Background

Group A *Streptococcus* (GAS) is a major component of human pharyngeal microbiota. However, GAS is associated with a wide spectrum of invasive diseases, such as pharyngitis, suppurative skin inflammation, scarlet fever, rheumatic fever and, in rare cases, streptococcal toxic shock syndrome (STSS). A mutation in the Control of Virulence Sensor gene (*covS)*, which is part of a 2-component control system in GAS, is thought to be involved in the onset of STSS [1]. Yet, this mechanism alone is insufficient to explain all cases of STSS. Moreover, the detailed pathogenic mechanism of STSS remains unclear. The serotypes of GAS strains are mainly characterized by two protein antigens expressed on the bacterial surface: M-type antigens, which are determined by the sequence of the *emm* gene encoding M protein; and T-type antigens, which are classified based on serological testing of enzyme-treated bacterial cells. Currently, most GAS strains associated with STSS worldwide have been classified as M1 T1 [2]. The M3-type strains are also frequently isolated from patients with STSS. The number of cases of STSS caused by M89- or M4-type strains is increasingly common in the United Kingdom and Australia [3, 4]. However, M-type antigen expression and pathogenicity are not necessarily correlated and therefore other factors should be considered to define the pathogenicity of GAS.

Several genome analyses of GAS have revealed that approximately 20% of its genome is composed of prophage genomes. These prophages encode various virulence factors, such as superantigens and DNases. Therefore, lateral transfer of virulence genes is associated with their dissemination and virulence [5]. Moreover, some GAS genomes also contain GAS clustered regularly interspaced palindromic repeats (CRISPR)/CRISPR-associated (Cas) systems, which confer adaptive immunity against exogenic elements, including bacteriophages (phages). Therefore, exploration of the relationship between phage acquisition and CRISPR/Cas systems in GAS genomes may enable a better understanding of the evolutionary aspects of GAS pathogenicity.

CRISPRs are composed of several short repeat sequences of approximately 30–50 bp, separated by unique variable sequences. These sequences are widely distributed and present in 90% of archaea and 50% of eubacteria [6–8]. The CRISPR-based adaptive immune system functions in 3 stages; acquisition, expression, and interference [8–11]. In the acquisition stage, specific fragments of double-stranded DNA from a virus or plasmid are acquired at the leader end of a CRISPR array in the bacteria host DNA [8–10]. Therefore, spacer sequences may provide a historical perspective on foreign DNA exposure, and thereby are useful as an indicator of their evolution [12].

Phages, viruses that infect eubacteria, play crucial roles in regulating bacterial ecology, diversity, and virulence. For example, phage-derived toxin genes, such as *sea*, *seg2*, *sek2*, and *sak,* are expressed in *Staphylococcus aureus* following prophage induction [13]. Enterohemorrhagic *Escherichia coli* also contain more than 20 prophage or prophage-like regions in its genome, and a Shiga toxin-encoded gene (*stx*) is found in Stx prophage [14]. Thus, phages can act as carriers of virulence-related genes, and the integrated phage genome can influence genome diversification in bacteria, leading to the emergence of pathogenic strains. A previous study conducted by us, using 13 sequenced GAS genomes, demonstrated that GAS CRISPR spacers target phages and that a small number of spacers may be limiting certain phage infections. In other words, being subjected to phage infections may lead to spacer acquisition in survivors [15]. However, 32% of the target sequences in GAS spacers were not included in the database, suggesting the existence of unknown phage, plasmid, ICE or other sequences, although some phylogenetic relationships of known prophages were predicted. Therefore, additional prophage analysis should help to further elucidate CRISPR interactions in GAS, as more complete genome information on GAS is released. In this study, we investigated the characteristics of two genomic elements, prophages and CRISPR, in 118 GAS genomes. Three hundred and seventy-three prophages found in these 118 strains were classified into 14 groups, based on integration site. Correlation between the number, or diversification, of prophages and the phylogenetic relationships of GAS was analyzed.

## Results

### Determination of CRISPR/*cas* loci and spacer sequence clustering

**Fig. 1:**
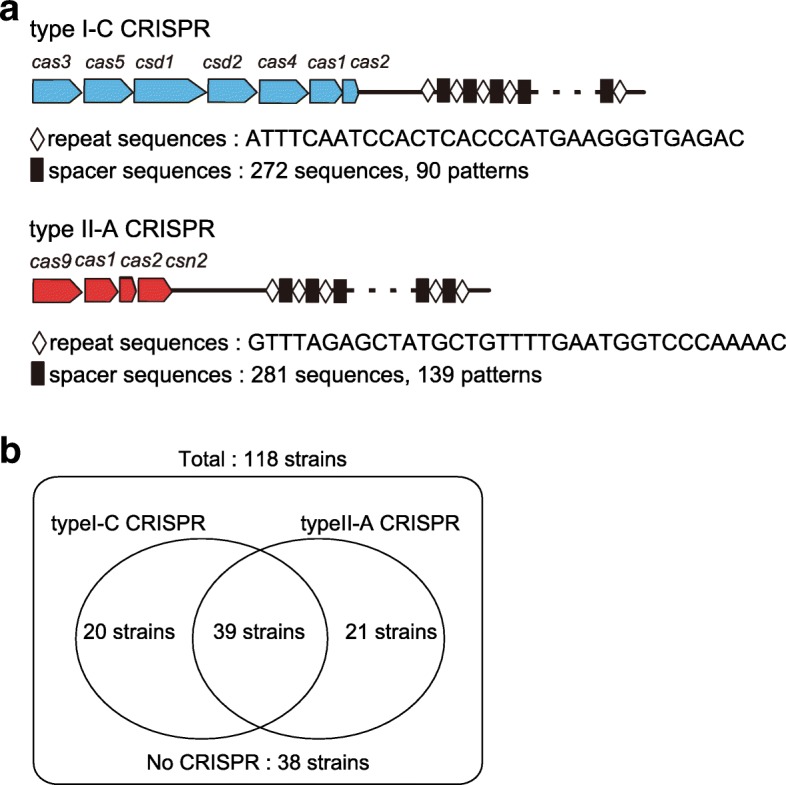
Genetic organization of type I-C and type II-A CRISPR/*cas* in 118 GAS strains. **a** *cas* genes are indicated in blue in type I-C and red in type II-A CRISPR/*cas*. Open diamonds and filled boxes indicate CRISPR repeats and spacers, respectively. **b** The distribution of 2 CRISPR types in 118 GAS strains represented in a Venn diagram. CRISPR, clustered regularly interspaced short palindromic repeats; GAS, Group A *Streptococcus*

CRISPR/*cas* loci in the 118 GAS strains were classified as either type I-C or type II-A (Fig. 1a). Type I-C and II-A CRISPR/*cas* corresponded to CRISPR2 and CRISPR1, respectively [15]. Both types were found in 39 strains and one was found in 41 strains, whereas no CRISPR locus was observed in 38 strains (Fig. 1b). Two hundred and seventy-two spacers were found in type I-C and 281 in type II-A CRISPR/*cas*. These spacers were clustered into 90 and 139 non-redundant unique groups in type I-C and II-A CRISPR/*cas*, respectively (Fig. 1a, Additional file 1: Tables S1 and S2).

### Clustering of GAS prophages and their phylogenetic relations

**Fig. 2:**
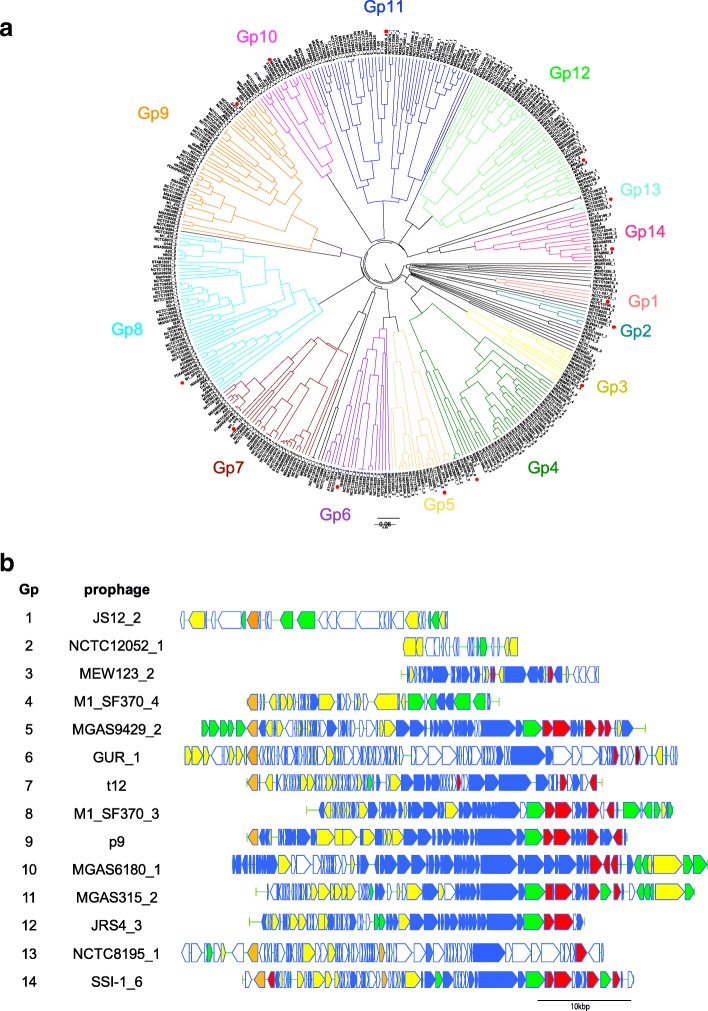
373 prophages in 118 hierarchically clustered strains and the genetic organization of 14 prophage groups. **a** Branches are color-coded by the corresponding prophage groups. The strains with red dots are representatives of prophage groups. **b** Genetic organization is shown for 14 representatives. CDSs are indicated by arrows and color-coded as follows; orange for integrase, yellow for replication and transcription-associated genes, green for metabolism-associated genes, red for virulence-associated genes, and blue for phage structural genes. CDSs with unknown function are indicated by open arrows

The number of prophages was predicted to be 373 in 118 genomes (Additional file 2); with an average of 3.16 phages per genome. MGAS10394 presented the highest number of prophages (8), whereas MGAS27061, MGAS23530, H293 and NCTC12068 did not harbor any prophages. The prophages varied in size from 5.7 to 96 kbp, with a mean of 46 kbp, and there were 7–132 CDSs per prophage, with a mean of 64 CDSs. The prophages of GAS were classified into 14 groups (Fig. 2a, b) via optimized threshold analysis (correlation distance < 0.421). Of the 14 groups, the streptococcal phages, t12, p9 and phi3396, were classified into prophage groups (Gps) 7, 9 and 14, respectively. Virulence genes encoded in each prophage were also found (Table 1, Additional file 1: Table S3). Gps 5, 8, 9, 10, 11, 12 and 14 encoded various kinds of virulence genes, such; as *speC, speH, speI, speK, slaA, ssa* and *sda*, whereas Gps 1, 2, 3, 4, 6 and 13 harbored no virulence genes. In contrast, *speA* was only found in Gps 7 and 14. In addition, some Gps shared integration sites (Table 1, Additional file 1: Table S3); [16]. For example, the integration sites of Gps 7, 10 and 11 and Gps 3 and 4 were *tmRNA* and *mutL*, respectively. Further analysis revealed that type II-A CRISPR regions served as an integration site for some prophages belonging to Gp 14. These encompassed four GAS strains with a defective type II-A CRISPR (SSI-1: 6 prophages, MGAS315: 6 prophages, STAB902: 6 prophages, and AP1: 5 prophages), in which phage integration was observed between *cas* genes and CRISPR array of type II-A CRISPR locus. These isolates had a larger number of prophages when compared to other GAS strains with type II-A CRISPR that appeared intact. A comparative analysis of the above Gps indicated that prophages in GAS may be classified based on the distribution of virulence genes, sequence similarity either at their 3′ and 5′ ends or phage structural regions and integration site.

**Table 1:** Summary of 14 phage groups in the GAS strains

Gp	Known virulence-related genes	Number of prophages	CDSs		Integration site
			min	max	
1	–	4	17	56	
2	–	5	10	18	
3	–	10	22	97	*mutL*
4	–	38	13	57	*mutL*
5	*ssa*, *speC*, *speH, speI*	18	55	91	dTDP-glucose-4,6,-dehydratase
6	*–*	20	23	132	
7	*speA*	32	29	87	*tmRNA, recX,* dTDP-glucose-4,6,-dehydrogenase
8	*ssa, speC, speH, speI*	49	29	96	DNA-binding protein HU
9	*ssa, sda*, *speH, speI*	45	44	78	*tRNAser*, dTDP-glucose-4,6,-dehydratase, Putative-gamma-glutamylkinase
10	*speC*, *speK, slaA*	17	53	84	*tmRNA*
11	*speC, slaA, speK, speH, speI, ssa*	48	24	96	Dipeptidase, *tmRNA*
12	*sda*, s*peC*, *slaA, speK*	47	21	130	Dipeptidase, *recX*, Promoter of *yesN*
13	*–*	3	61	69	
14	*speA, speC, ssa*	15	53	124	CRISPR type II-A, RNA helicase

*CDS* coding sequence, *CRISPR* clustered regularly interspaced short palindromic repeats, *GAS* Group A *Streptococcus*, *Gp* prophage group

### Relationship between spacer sequences and prophages

**Fig. 3:**
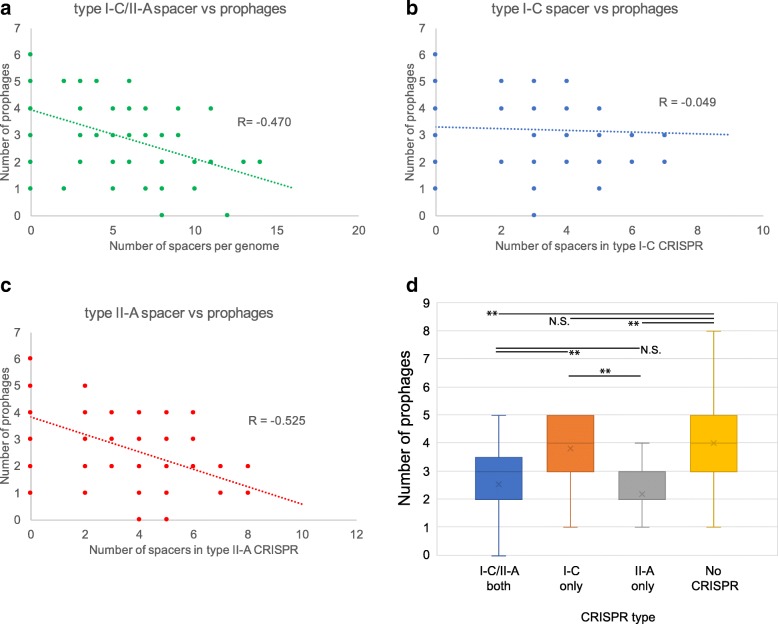
Correlation between the number of spacer sequences and prophages. **a**-**c** The number of spacers in each strain is plotted against the number of prophages in scatter graphs for types I-C and II-A (**a**), type I-C (**b**), and type II-A CRISPR/*cas* (**c**). Spearman’s rank correlation coefficients are indicated as R values. **d** The number of prophages in each GAS genome harboring various type of CRISPR are shown using box plots.“×” indicates median of the number of prophages. Two asterisks indicate statistical significance at *p* < 0.01, and N.S. indicates non-significance

We investigated correlation between spacer sequences and prophages identified in this study. We found that the number of spacers were negatively correlated with prophages in GAS strains (Fig. 3a-c). There was a significant negative correlation between the number of spacers and prophages in type II-A CRISPR (R = − 0.525). Moreover, the average number of prophages in strains with type II-A CRISPR or type I-C/II-A CRISPR was significantly lower than that in strains without CRISPR. By contrast, there were no significant differences between strains with type I-C CRISPR and those without CRISPR (Fig. 3d). These findings indicated that: (1) phage rejection ability of CRISPR loci may be different in type I-C and type II-A CRISPR; and (2) type II-A CRISPR functions more effectively than type I-C CRISPR.

**Fig. 4:**
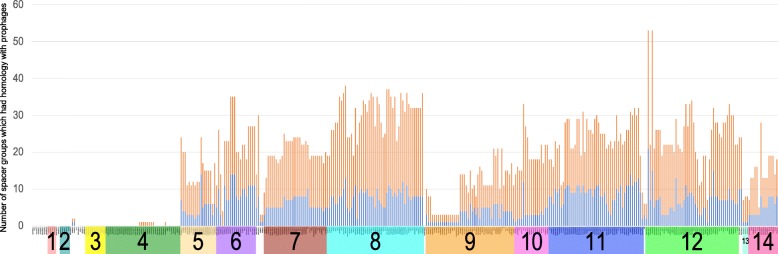
The number of spacers exhibiting nucleotide similarity with 14 prophage groups. The number of spacers exhibiting nucleotide similarity with the prophages in each strain is indicated in the bar chart. The strains are horizontally listed according to prophage groups. The spacers in type I-C and II-A CRISPR/*cas* are colored in blue and orange, respectively

To elucidate functional differences between the two CRISPR, we further analyzed differences in the spacer sequences of GAS strains. Of the 229 spacer sequence patterns (Additional file 1: Table S2), 204 (89.1%) exhibited similarity (bit scores ≥50) with other GAS prophage regions, including 3 phages previously isolated from GAS (Additional file 1: Table S4), and 1 showed homology with that of the prophage *Streptococcus agalactiae* str. ILRI005. This criterion using bit scores roughly correlates to 2 nucleotide differences over the average spacer length of 30 nucleotides, based on previous report [17] with experimental proof using *Streptococci*. In this study, the lowest bit score was 52.8, which roughly correlates to a perfect match of 28 nucleotides, or less than 1 nucleotide difference over the 34 nucleotides. The remaining 24 (10.5%) sequences were not homologous to any other sequences in the National Center for Biotechnology Information database. This number was lower than that observed in our previous study, indicating the identification of novel prophages in this study. We further investigated the relationship between spacers and Gps. Type II-A spacer groups accounted for more than 69% of the total number of homologous groups between spacers and prophages (Fig. 4), suggesting that type II-A CRISPR may contribute to the elimination of invading phages. This observation is consistent with the correlation between the number of spacers and prophages (Fig. 3), indicating that the phages are eliminated by the CRISPR/Cas system of GAS, but that such elimination efficiency may affect the number of prophages in the GAS genome. However, there was no significant difference between Gps targeted by spacers derived from each CRISPR type (Additional file 1: Table S5).

Notably, no spacer group exhibited homology with prophages from Gps 1, 2, or 3, or subsets of Gp 4. Prophages of Gps 3 and 4 integrated in the region of *mutL*, which encodes the DNA mismatch repair protein, *MutL*. We further inspected the gene composition of Gp 3 and 4 prophages. Results indicated that, these prophages lacked phage structural genes, such as capsid protein and tail protein. These findings indicated that some phage groups with specific integration site are not targeted by the CRISPR.

### Comprehensive analysis of GAS strains and prophage groups

**Fig. 5:**
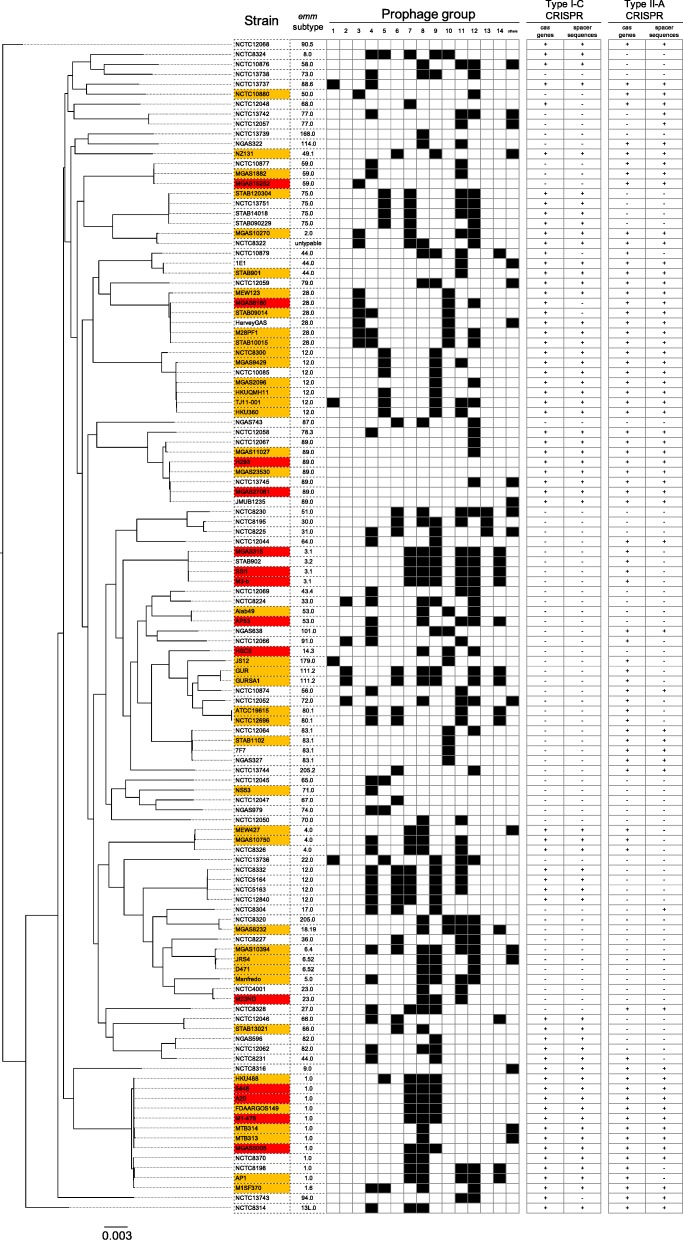
Phylogenetic analysis of 118 GAS strains with clinical symptom, M-type, prophage group, and CRISPR/*cas* data. The maximum likelihood phylogenetic tree was constructed from 715 CDSs by the GTR substitution model, and is shown on the left. The strains with severe and mild symptoms are indicated by red and orange, respectively, and strains with asymptomatic or unknown symptoms are not colored. The presence and absence of prophages are indicated by filled and open boxes, respectively, for the 14 prophage groups listed horizontally. The presence and absence of *cas* genes and CRISPR are represented by “+” and “-”, respectively

Results of the phylogenetic analysis, M-type analysis, CRISPR/Cas analysis and prophage analysis were compared for possible associations (Fig. 5, Additional file 1: Table S6). The M-type analysis identified 51 M-type strains. In the phylogenetic analysis, the distribution of M-type antigens was relatively consistent with the phylogeny of GAS except for subsets of M12. Moreover, GAS strains with similar M-type antigens had similar Gp combinations as follows: M3 type strains: Gps 7, 8, 9, 11, 12, and 14; M28 type strains: Gps 3 and 10; M12 type strains: Gp 9; and M1 type strains: Gp 8. Prophages were also conserved in multiple M-type strains; Gp 7 prophages were conserved in all M3, M4, M75 strains and in 75% of M1 strains; whereas Gp 9 prophages were conserved in all M3, M6, and M12 strains and in 50% of M1 strains. The 118 GAS strains used in this study were isolated from patients with a variety of clinical symptoms, and an attempt was made to elucidate the correlation between STSS and Gps. However, the Gps did not correlate with STSS. STAB902 isolated from a non-invasive superficial cutaneous infection [18] harbored the same set of prophages as MGAS315 and SSI-1. By contrast, there were no prophages in the STSS strains, H293 and MGAS27061.

## Discussion

The CRISPR/Cas system is widely conserved in archaea and bacteria. Moreover, type I-C CRISPR is widely conserved and considered to be an original model of CRISPR, whereas, type II CRISPR is only conserved in bacteria. Among them, type II-A CRISPR is mainly conserved in Firmicutes, including GAS [19]. Nozawa et al., reported that GAS contained both type I-C and type II-A CRISPR [15]. In the present study, we did not detect any novel CRISPR loci in the GAS genome. Therefore, the CRISPR locus in GAS may mainly consist of these two CRISPR systems.

In the present study, we inferred that the CRISPR/Cas system was functioning in the GAS genome, which was consistent with the findings of a previous study [15]. Moreover, we proposed that the main function of type II-A CRISPR is the elimination of phages, which protects the GAS genome more effectively than type I-C. Correlation between the number of type I-C spacer sequences and the number of prophages was relatively weak compared with that in type II-A (Fig. 3). This is inconsistent with the findings of our previous study [15]. Only 13 strains were used in our previous study, and most strains were isolated in the United States. Therefore, we speculated that the difference may be due to increased variation in M type and the isolated region used in this study.

We observed a significant difference in the number of prophages between type II-A CRISPR and no CRISPR strains. Therefore, our hypothesis that type II-A CRISPR may plays an important role in eliminating foreign DNA from GAS, was consistent with the findings of a study on *S. mutans* [20]. Therefore, we reached the conclusion that type II-A CRIPSR may be the main source of phage rejection and elimination in the GAS genome.

GAS prophages were classified into 14 groups based on the presence or absence of ortholog clusters (Fig. 2). Prophage clustering raises issues due to the diversity of accessory genes and mosaic structures of prophages. The mosaic structure of prophages may induce genomic rearrangement via a ~ 5 kb sequence located near the 3′ terminus, as previously reported [21]. Therefore, only DNA alignment has been used to elucidate phylogenetic relationships in GAS prophages [16]. In this study, we used the GAS ortholog clusters for the clustering of prophages, and examined sequence similarities in each prophage group (Additional file 3: Figure S1 A-N). The sequence similarities of prophages in GAS can be roughly classified into two patterns: (1) the regions around 5′ and 3′ termini where genes encoding enzymes, such as integrase, and various kinds of virulence genes are conserved and (2) the central part of prophages encoding structural proteins is conserved. For example, 5′ and 3′ terminus regions tend to be conserved in Gps 4 and 10, whereas, regions with genes encoding structural proteins tend to be conserved in Gps 5, 6 and 14. These findings suggest the possibility of elucidating rearrangement regions systematically by examining prophage sequences and groups. A similar clustering method was used in a study on *E. coli* prophages, where *E. coli* Gps correlated with the shiga-toxin gene type [22]. Although, we were unable to find a relationship between GAS Gps and clinical symptoms, GAS Gps correlated with the integration site. Moreover, GAS strains harboring prophages that targeted the same genes that the integration site did, were not necessarily phylogenetically related. Therefore, we suggest that these genes tend to be the targets of phage integration.

The spacer sequences did not show homology with the Gps 1, 2, 3, and a majority of 4, characterized by *mutL* integration sites (Fig. 4). In previous reports, prophages such as SF370_4, Manfredo_5 and MGAS15252_1 were classified into streptococcal phage-like chromosomal islands (SpyCI) [16, 23]. Gps 3 and 4 lack structural proteins but harbored genes encoding DNA mismatch repair, multidrug efflux and Holliday-junction resolvase. Therefore, we speculated that Gps 3 and 4 consisted of SpyCI, since these characteristics are specifically found in SpyCI. As reported previously [24], SpyCI are believed to have originated from defective prophages. Also, the presence of a SpyCI in GAS correlates with a higher mutation rate and UV sensitivity compared to strains lacking SpyCI [25]. However, there have been no reports indicating that SpyCI are not targeted by CRISPR. One possible reason for these Gps not being recognized by CRISPR is that the spacer’s target region is absent or has mutated during evolution. Reportedly, deletion of spacer sequences may occur frequently [26]. Therefore, it may be speculated that GAS lacks spacers targeting these prophages. Furthermore, other non-CRISPR mechanisms, such as restriction-modification (R-M), toxin-antitoxin, and abortive infection systems may play a stronger role at phage rejection and elimination in GAS as well as CRISPR/Cas systems. For example, SF370 and MGAS10394 have type I and II R-M systems, respectively [27, 28]. Nonetheless, these results suggest that acceptance of foreign DNA by GAS may be related to the integration site.

Additionally, the study indicated that 3 different data types, M-type, spacer sequences, and prophage groups, strongly correlated with GAS phylogenetic relationships (Fig. 5 and Additional file 1: Table S6). These findings suggest that phage integration into the GAS genome is dependent on genomic characteristics of GAS such as M type antigens. For example, a previous study indicated that almost all M12 strains harbored Gp 9 prophages, and that this phage integration may have occurred before the 1950s [29]. Similarly, it has been reported that MGAS315_6, in prophage Gp 9, was integrated into the GAS genome before 1920 [30]. Considered together, Gp 9 prophages may have been integrated into the GAS genome before STSS and other streptococcal diseases emerged. In contrast, Davis et al., suggested that HKU360_2 (Gp 11) and HKU360_4 (Gp 5) triggered the expansion of M12 strains causing scarlet fever in Hong Kong in 2011 [31]. Although we did not find a clear correlation between Gps and STSS in this study, it may be possible to estimate pathogenic and non-pathogenic prophage groups via further analyses of the clustering/classification patterns of GAS prophages.

The strong correlation between the 3 data types, M-type, spacer sequences and Gps may be useful for estimating genetic characteristics and/or phylogenetic relationships of GAS. This approach was previously known as “CRISPR typing” and used in studies on *Salmonella enterica* [32], *Mycobacterium tuberculosis* [33], *Yersinia pestis* [34] and *Corynebacterium diphtheriae* [35]. For example, *S. enterica* strains were classified via conventional virulence gene analysis together with CRISPR typing [32]. Additionally, epidemiological trials inferring region-specific strains and estimating their dissemination routes have been conducted with *Y. pestis* [34].

## Conclusions

The current study classified prophages found in 118 GAS strains and investigated correlation between spacer sequences and prophages. Results of phylogenetic and M-type analyses suggested that type II-A CRISPR may play an important role in eliminating phages, and that the integration site may be the deciding factor in acceptance of foreign DNA by GAS. It was also observed that clustered Gps correlated with M-type and their integration site. The present study provides novel information that will help analyze GAS phylogeny in detail based on M-type, spacer sequences and prophage distribution.

## Methods

### GAS genome sequences

The complete genome sequences of 118 GAS strains were downloaded from the PATRIC genome online database (http://www.patricbrc.org) (Additional file 1: Table S7).

Only complete genomes were analyzed as draft genomes had a lower predicted number of prophages (Additional file 3: Figure S2) and this would affect the results.

### Phylogenetic analysis of GAS strains

Protein-coding sequences (CDSs) shared among all strains were identified via the rapid large-scale prokaryote pan genome analysis (ROARY) [36], with default parameters, and were defined as core CDSs. Nucleotide sequences of all core CDSs were concatenated in each strain, and aligned using MAFFT-v7.149b [37]. A maximum likelihood-based phylogenetic tree was constructed from concatenated core CDSs using Model Generator-v851 [38], and Randomized Axelerated Maximum Likelihood (RAxML) ver. 7.2.8 [39] with 100 bootstrap replicates.

### Identification of M-type strains

All CDSs were searched using BLASTN [40] against the *emm* database in the Centers for Disease Control and Prevention (CDC, ftp://ftp.cdc.gov/pub/infectious_diseases/biotech/emmsequ/). CDS with a perfect match to a particular *emm* gene in the database was considered to be the *emm* gene of that strain, and the corresponding M type was assigned.

### Prediction and clustering of CRISPR/*cas* loci

CRISPR loci were predicted using the CRISPR Recognition Tool ver. 1.2 [41] with default parameters, and the candidates were manually verified. All spacers were clustered as described previously [17], and the representatives of all clusters were considered to be non-redundant unique spacers. The *cas* genes in each strain were classified based on *cas* gene arrays in strain SF370.

### Prediction and clustering of prophages

Prophages in each genome were predicted using PhiSpy ver. 2.2 [42], and prophage sequences were re-annotated using Prokka ver. 1.11 [43]. The prophages were then clustered using GetHomologues [44] with default parameters. The genetic organization of prophage for each cluster was visualized in silico with Molecular Cloning Genomics Edition (IMC-GE) ver. 7.09 (In Silico Biology, Kanagawa, Japan) [45]. The distance matrix was calculated from the binary information of presence or absence of prophages, and used to construct a dendrogram based on complete-linkage hierarchical clustering with 10,000 bootstrap replicates using *hclust* in R [46]. The similarity of prophage sequences in each prophage group was examined using GenoMatcher [47].

### Statistical analyses

Maharabinos distance was calculated to examine the correlation between the number of spacers and prophages. Outliers were eliminated according to statistical significance (*P* < 0.05). The remaining spacers and prophages were examined by estimating Spearman’s rank correlation coefficient using R [46]. Differences in the number of prophages between two groups were examined using the Mann-Whitney *U* test: the strains harboring type I-C and II-A CRISPR/*cas*, the strains harboring type I-C only, the strains harboring type II-A only, and the strains without any CRISPR/*cas*. Statistical significance was set at *P* < 0.05.

All data generated or analyzed during this study are included in this article and additional files.

## Additional files

Additional file 1: **Table** **S1.** A Spacer sequneces of type I-C CRISPR identified from all 118 strains. B Spacer sequneces of type II-A CRISPR identified from all 118 strains. **Table S2.** A Clustered spacer sequence groups of type I-C CRISPR. Criteria of clustering is as mentioned above. B Clustered spacer sequence groups of type II-A CRISPR. Criteria of clustering is as mentioned above. **Table S3.** Detailed information of prophages. **Table S4.** Prophages targeted by each spacer sequences. **Table S5.** Correlation between spacers and prohpage groups. Spacers derived from each CRISPR are indicated in the column and prophages are indicated in the row. Spacers that exibited similarity with prophage groups are indicated as black boxes. **Table S6.** Result of the possession of *cas* genes and CRISPR arrays in each strain. **Table S7.** Strains used in this study. (XLSX 183 kb)

Additional file 2: Three hundred seventy three prophage sequences predicted in 118 GAS genomes. (TXT 16655 kb)

Additional file 3: **Figure S1.** Mutual synteny plot of GAS prophages in each prophage group. A-N: The numbers on x-axes correspond to the numbers indicated in the table on the right. GAS; Group A *Streptococcus*. **Figure S2.** Number of prophages predicted in draft GAS genomes and complete GAS genomes. Box plot of the number of prophages of draft GAS genomes and complete GAS genomes. The boxes indicate the medians and 25th–75th percentiles. Whiskers indicate 5th to 95th percentiles and outliers are indicated by the closed circle. *P*-value was determined by student’s *t*-test. (PPTX 27905 kb)

## Abbreviations

CDS: Coding sequence
CRISPR: Clustered regularly interspaced short palindromic repeats
GAS: Group A *Streptococcus*
Gp: Prophage group
STSS: Streptococcal toxic shock syndrome
