# Supplementary material for: Phylogenetic relationship of prophages is affected by CRISPR selection in Group A Streptococcus
Source: BMC Microbiol. 2019 Jan 28;19:24. doi: 10.1186/s12866-019-1393-y (PMC6348661; doi:10.1186/s12866-019-1393-y)
Supplement: Supplementary file 3 — Figure S1. Mutual synteny plot of GAS prophages in each prophage group. A-N: The numbers on x-axes correspond to the numbers indicated in the table on the right. GAS; Group A Streptococcus. Figure S2. Number of prophages predicted in draft GAS genomes and complete GAS genomes. Box plot of the number of prophages of draft GAS genomes and complete GAS genomes. The boxes indicate the medians and 25th–75th percentiles. Whiskers indicate 5th to 95th percentiles and outliers are indicated by the closed circle. P-value was determined by student’s t-test. (PPTX 27905 kb) [file 12866_2019_1393_MOESM3_ESM.pptx]

## Slide 1
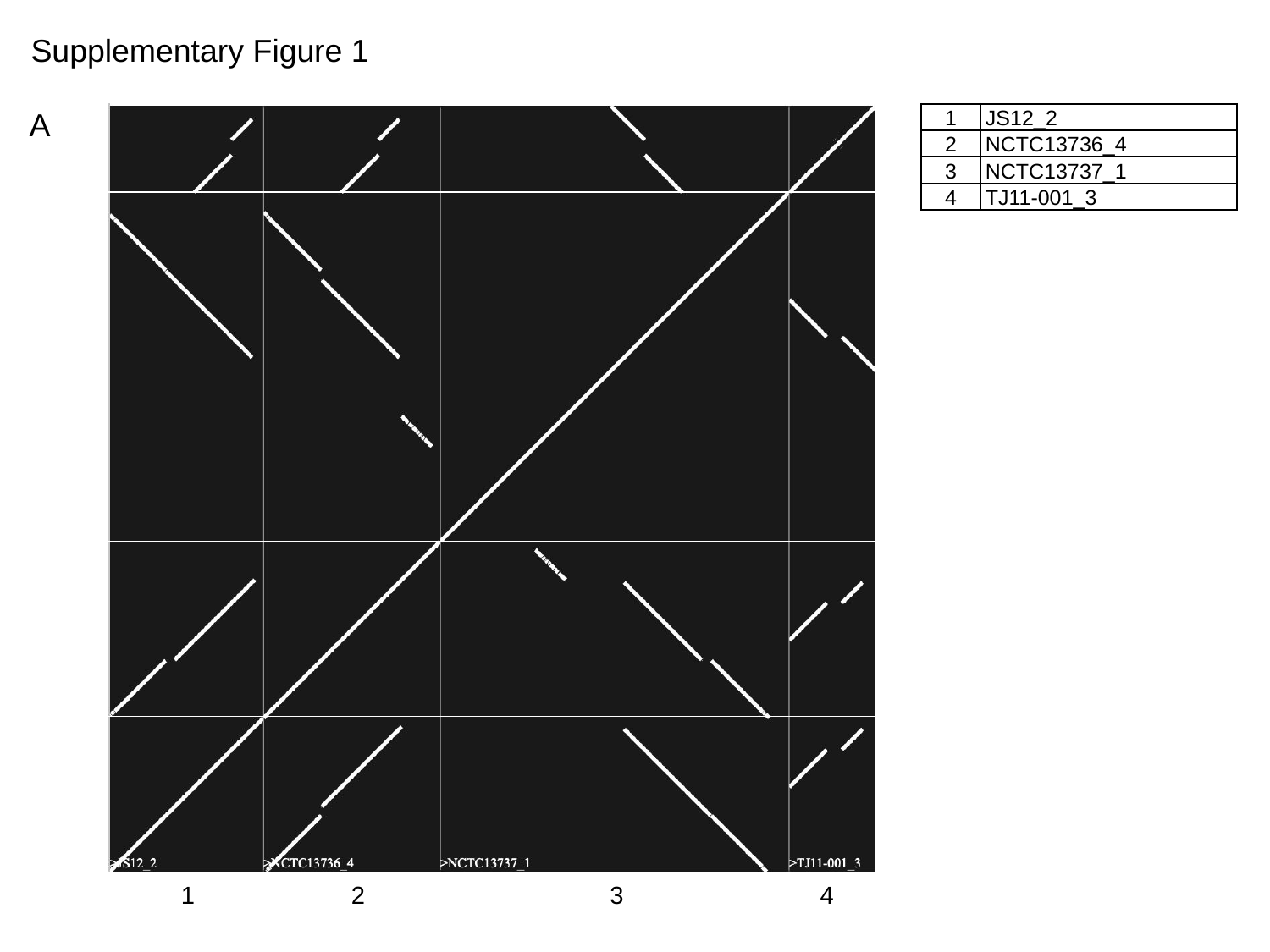

Supplementary Figure 1
A
| 1 | JS12\_2 |
| --- | --- |
| 2 | NCTC13736\_4 |
| 3 | NCTC13737\_1 |
| 4 | TJ11-001\_3 |
3
1
2
4

## Slide 2
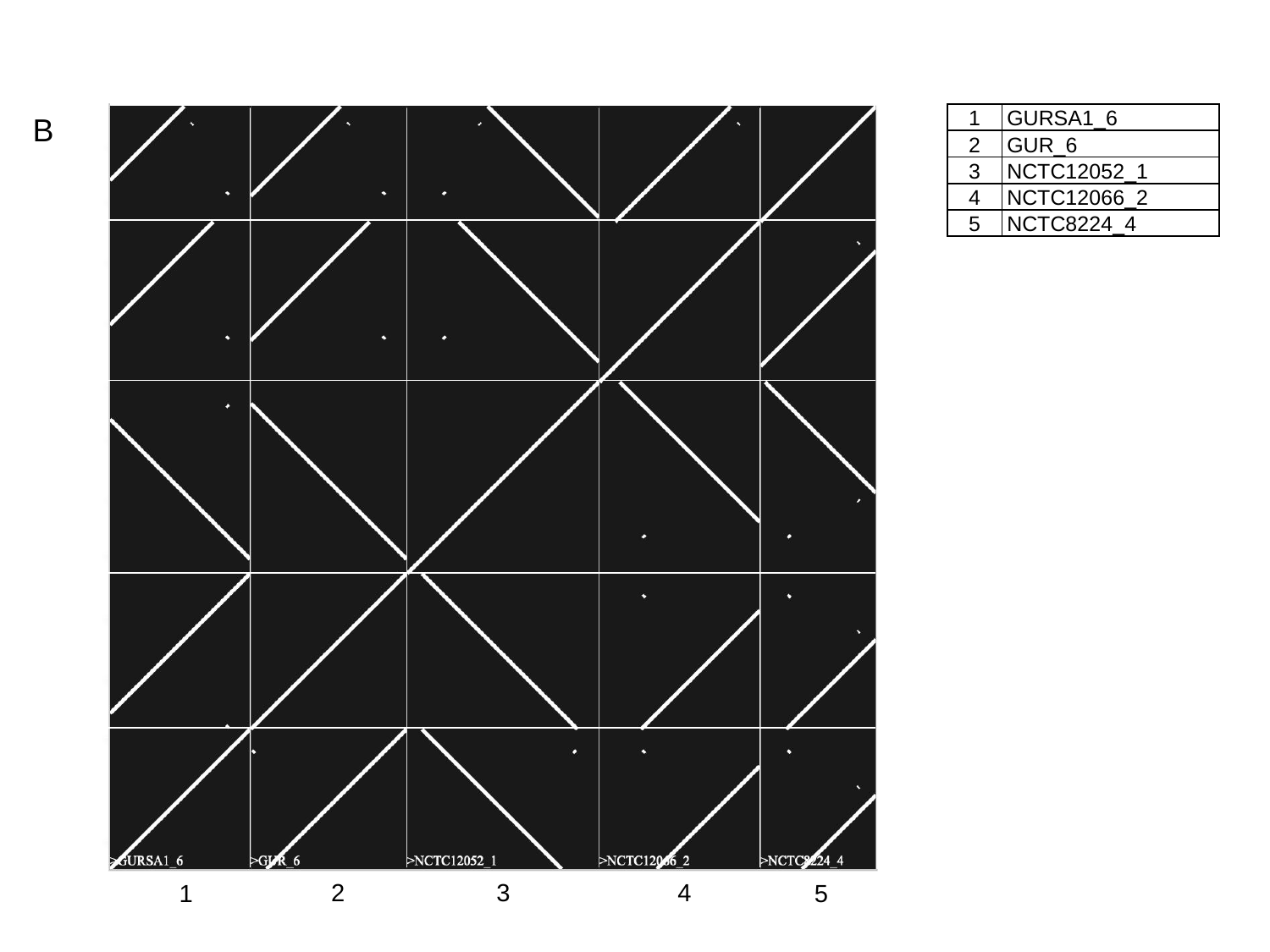

B
| 1 | GURSA1\_6 |
| --- | --- |
| 2 | GUR\_6 |
| 3 | NCTC12052\_1 |
| 4 | NCTC12066\_2 |
| 5 | NCTC8224\_4 |
3
2
4
1
5

## Slide 3
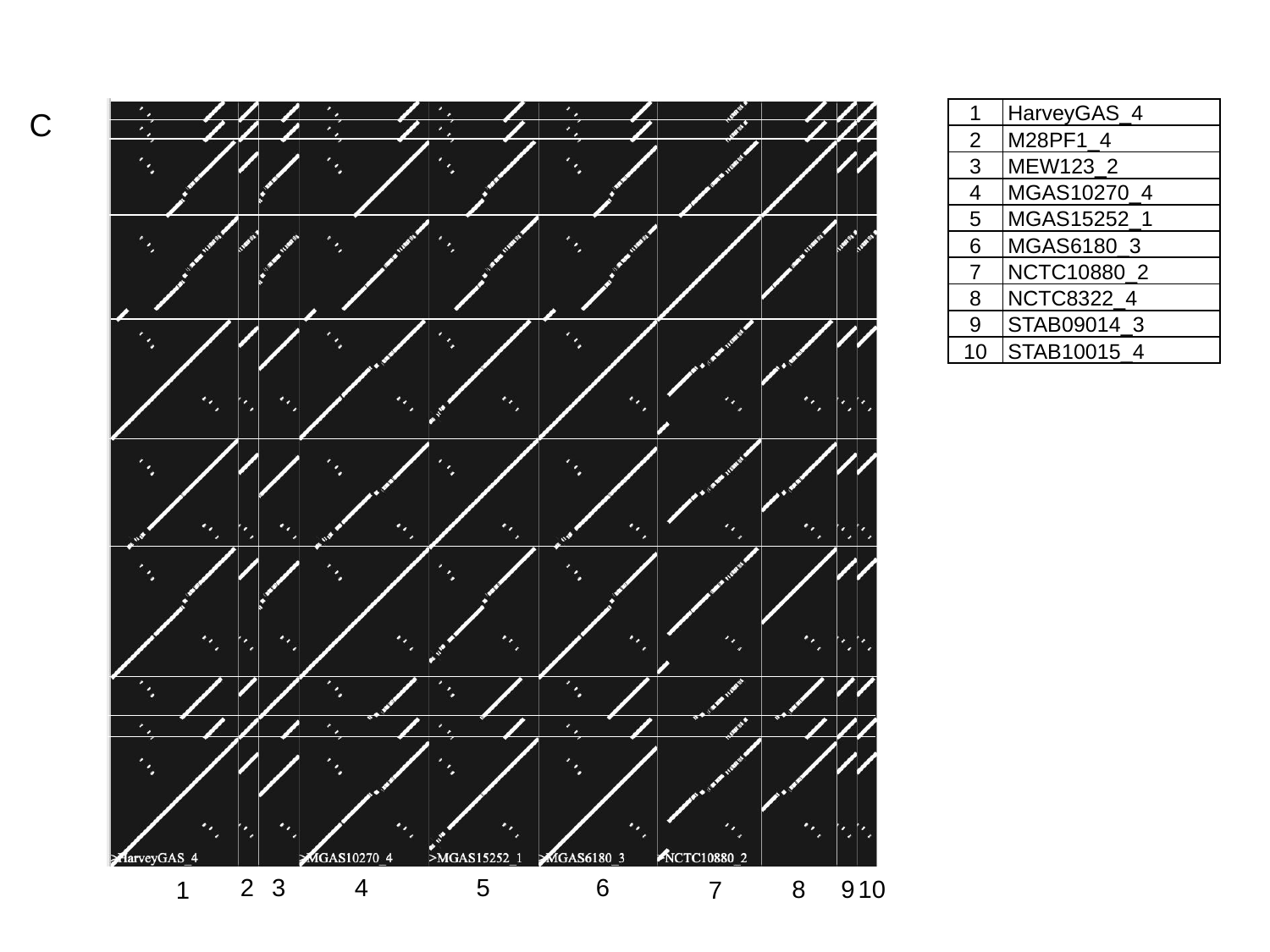

C
| 1 | HarveyGAS\_4 |
| --- | --- |
| 2 | M28PF1\_4 |
| 3 | MEW123\_2 |
| 4 | MGAS10270\_4 |
| 5 | MGAS15252\_1 |
| 6 | MGAS6180\_3 |
| 7 | NCTC10880\_2 |
| 8 | NCTC8322\_4 |
| 9 | STAB09014\_3 |
| 10 | STAB10015\_4 |
5
2
3
4
6
9
10
8
7
1

## Slide 4
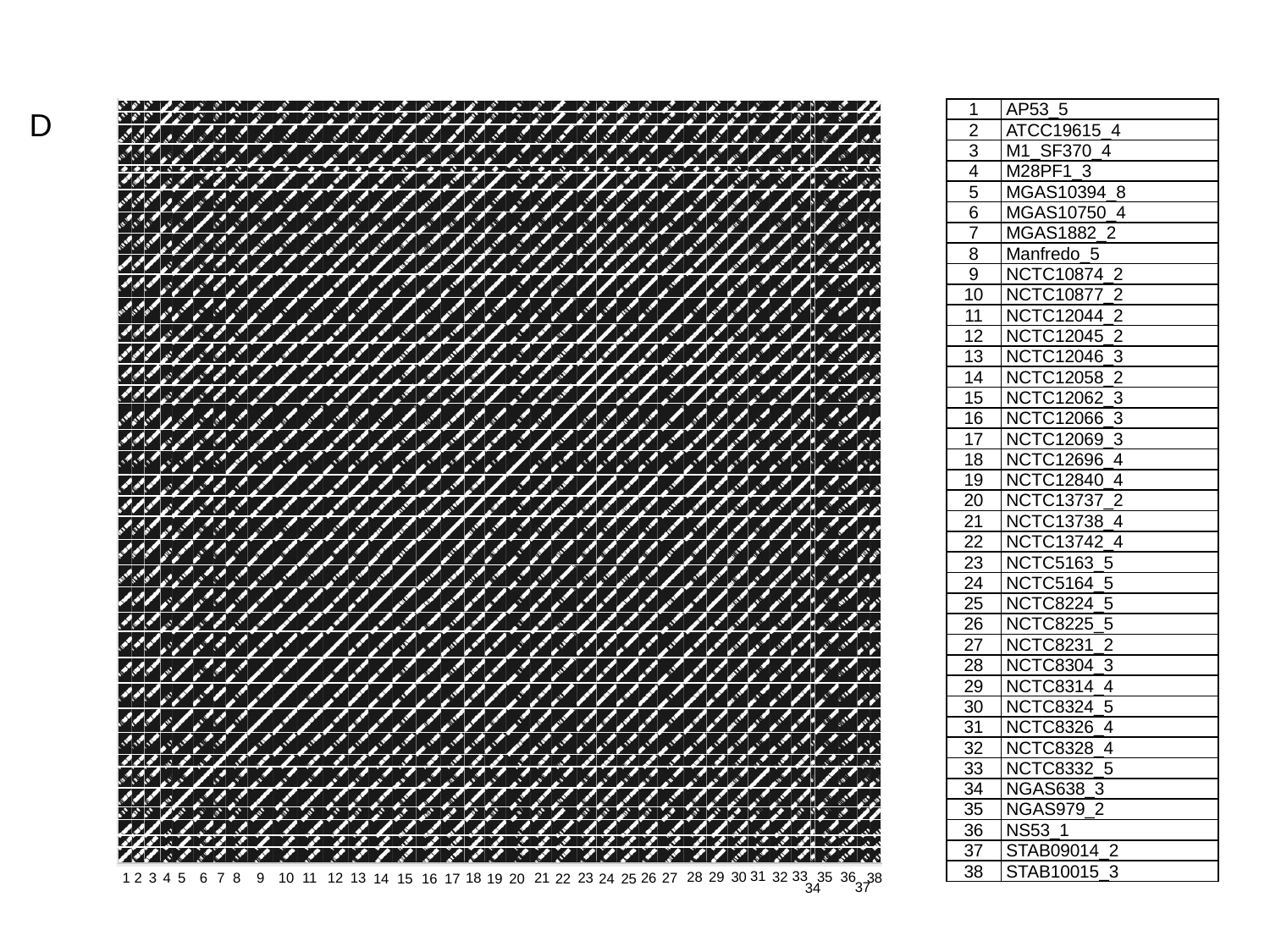

D
| 1 | AP53\_5 |
| --- | --- |
| 2 | ATCC19615\_4 |
| 3 | M1\_SF370\_4 |
| 4 | M28PF1\_3 |
| 5 | MGAS10394\_8 |
| 6 | MGAS10750\_4 |
| 7 | MGAS1882\_2 |
| 8 | Manfredo\_5 |
| 9 | NCTC10874\_2 |
| 10 | NCTC10877\_2 |
| 11 | NCTC12044\_2 |
| 12 | NCTC12045\_2 |
| 13 | NCTC12046\_3 |
| 14 | NCTC12058\_2 |
| 15 | NCTC12062\_3 |
| 16 | NCTC12066\_3 |
| 17 | NCTC12069\_3 |
| 18 | NCTC12696\_4 |
| 19 | NCTC12840\_4 |
| 20 | NCTC13737\_2 |
| 21 | NCTC13738\_4 |
| 22 | NCTC13742\_4 |
| 23 | NCTC5163\_5 |
| 24 | NCTC5164\_5 |
| 25 | NCTC8224\_5 |
| 26 | NCTC8225\_5 |
| 27 | NCTC8231\_2 |
| 28 | NCTC8304\_3 |
| 29 | NCTC8314\_4 |
| 30 | NCTC8324\_5 |
| 31 | NCTC8326\_4 |
| 32 | NCTC8328\_4 |
| 33 | NCTC8332\_5 |
| 34 | NGAS638\_3 |
| 35 | NGAS979\_2 |
| 36 | NS53\_1 |
| 37 | STAB09014\_2 |
| 38 | STAB10015\_3 |
33
31
29
32
35
30
28
36
27
11
8
7
9
38
3
4
5
12
1
6
23
10
18
26
2
13
21
16
24
19
22
14
17
25
20
15
37
34

## Slide 5
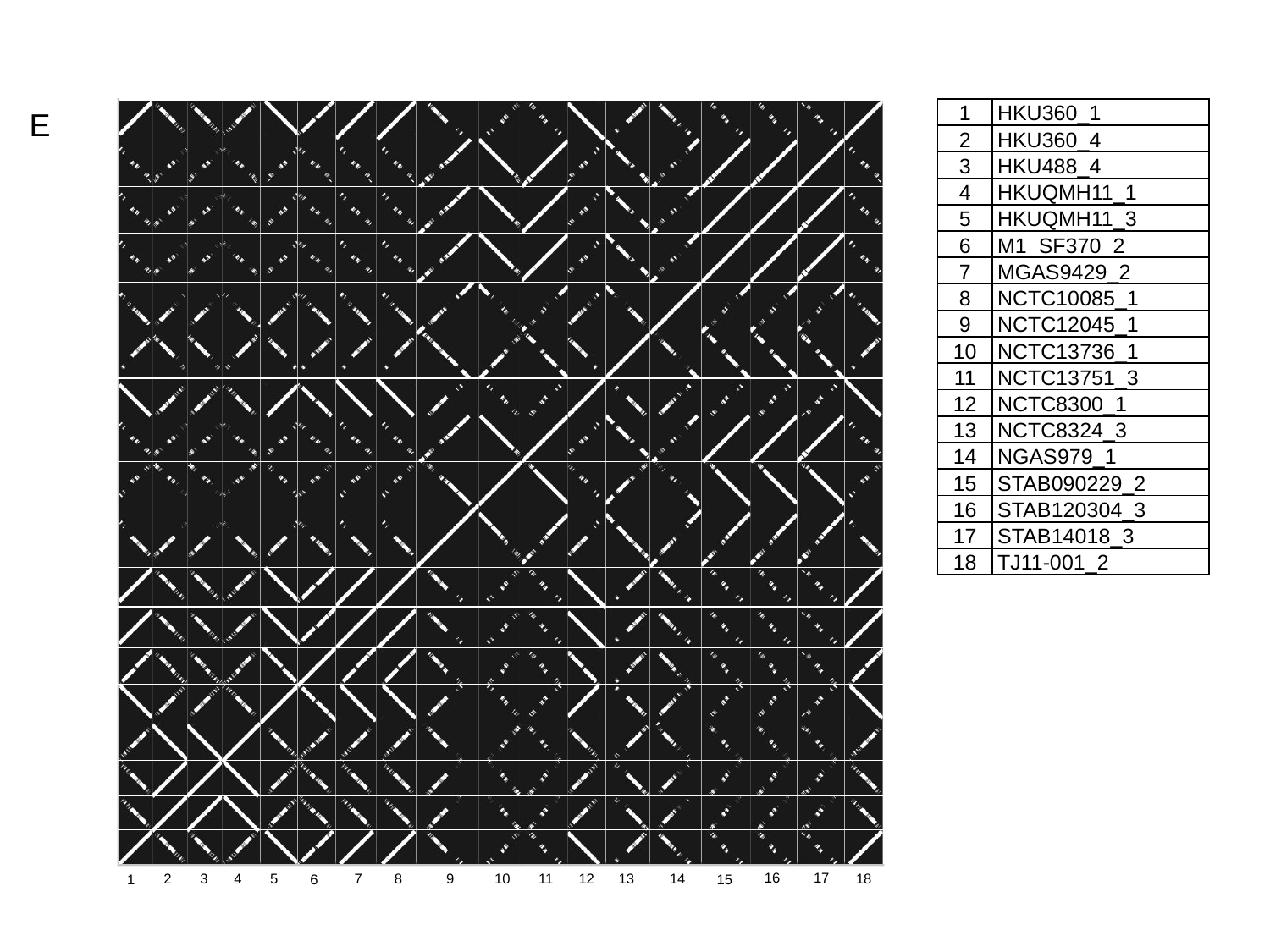

E
| 1 | HKU360\_1 |
| --- | --- |
| 2 | HKU360\_4 |
| 3 | HKU488\_4 |
| 4 | HKUQMH11\_1 |
| 5 | HKUQMH11\_3 |
| 6 | M1\_SF370\_2 |
| 7 | MGAS9429\_2 |
| 8 | NCTC10085\_1 |
| 9 | NCTC12045\_1 |
| 10 | NCTC13736\_1 |
| 11 | NCTC13751\_3 |
| 12 | NCTC8300\_1 |
| 13 | NCTC8324\_3 |
| 14 | NGAS979\_1 |
| 15 | STAB090229\_2 |
| 16 | STAB120304\_3 |
| 17 | STAB14018\_3 |
| 18 | TJ11-001\_2 |
17
16
13
18
11
10
2
12
3
4
5
14
8
7
9
15
6
1

## Slide 6
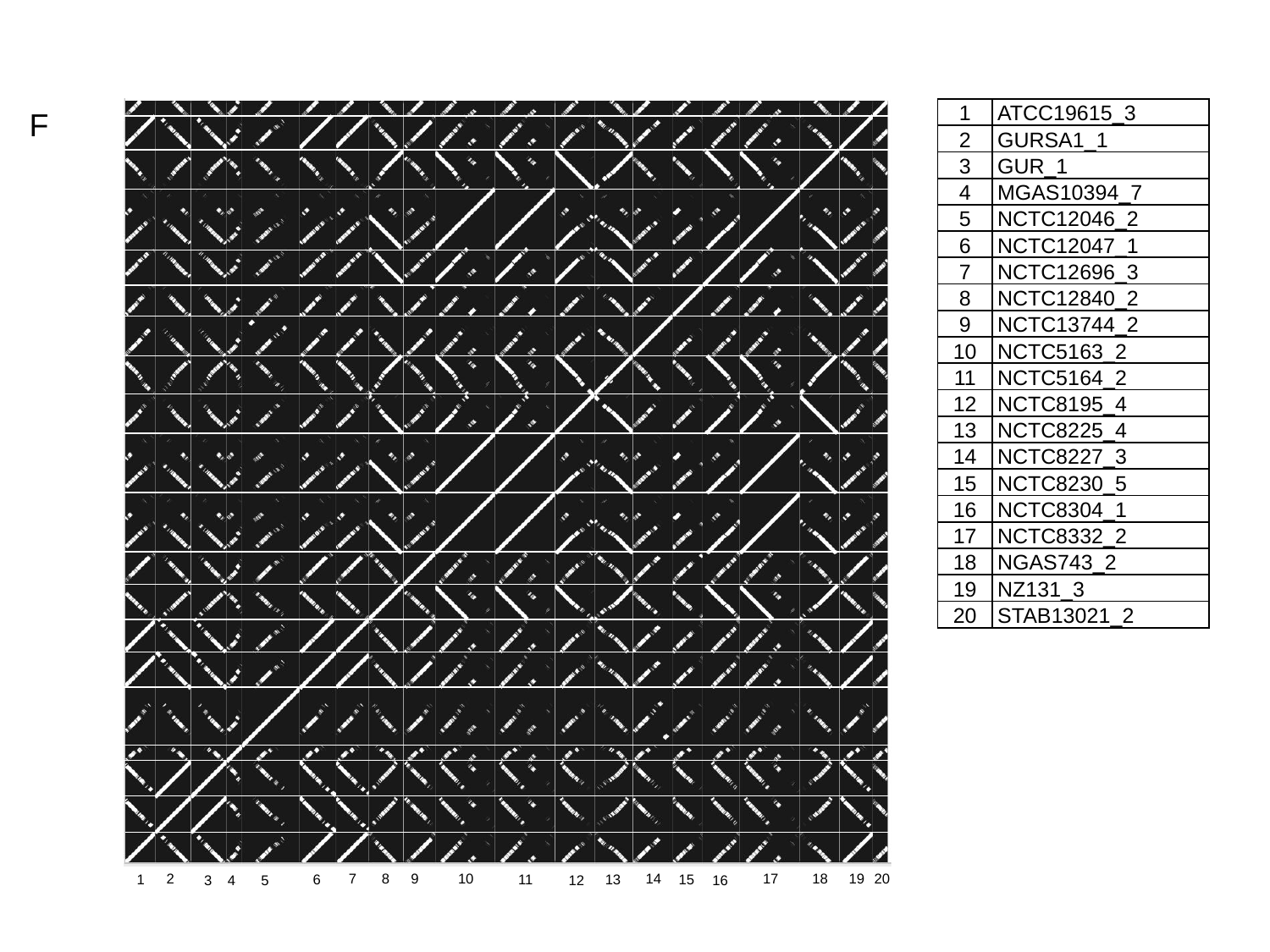

F
| 1 | ATCC19615\_3 |
| --- | --- |
| 2 | GURSA1\_1 |
| 3 | GUR\_1 |
| 4 | MGAS10394\_7 |
| 5 | NCTC12046\_2 |
| 6 | NCTC12047\_1 |
| 7 | NCTC12696\_3 |
| 8 | NCTC12840\_2 |
| 9 | NCTC13744\_2 |
| 10 | NCTC5163\_2 |
| 11 | NCTC5164\_2 |
| 12 | NCTC8195\_4 |
| 13 | NCTC8225\_4 |
| 14 | NCTC8227\_3 |
| 15 | NCTC8230\_5 |
| 16 | NCTC8304\_1 |
| 17 | NCTC8332\_2 |
| 18 | NGAS743\_2 |
| 19 | NZ131\_3 |
| 20 | STAB13021\_2 |
18
19
20
10
2
14
17
8
7
9
15
6
1
13
11
12
3
4
5
16

## Slide 7
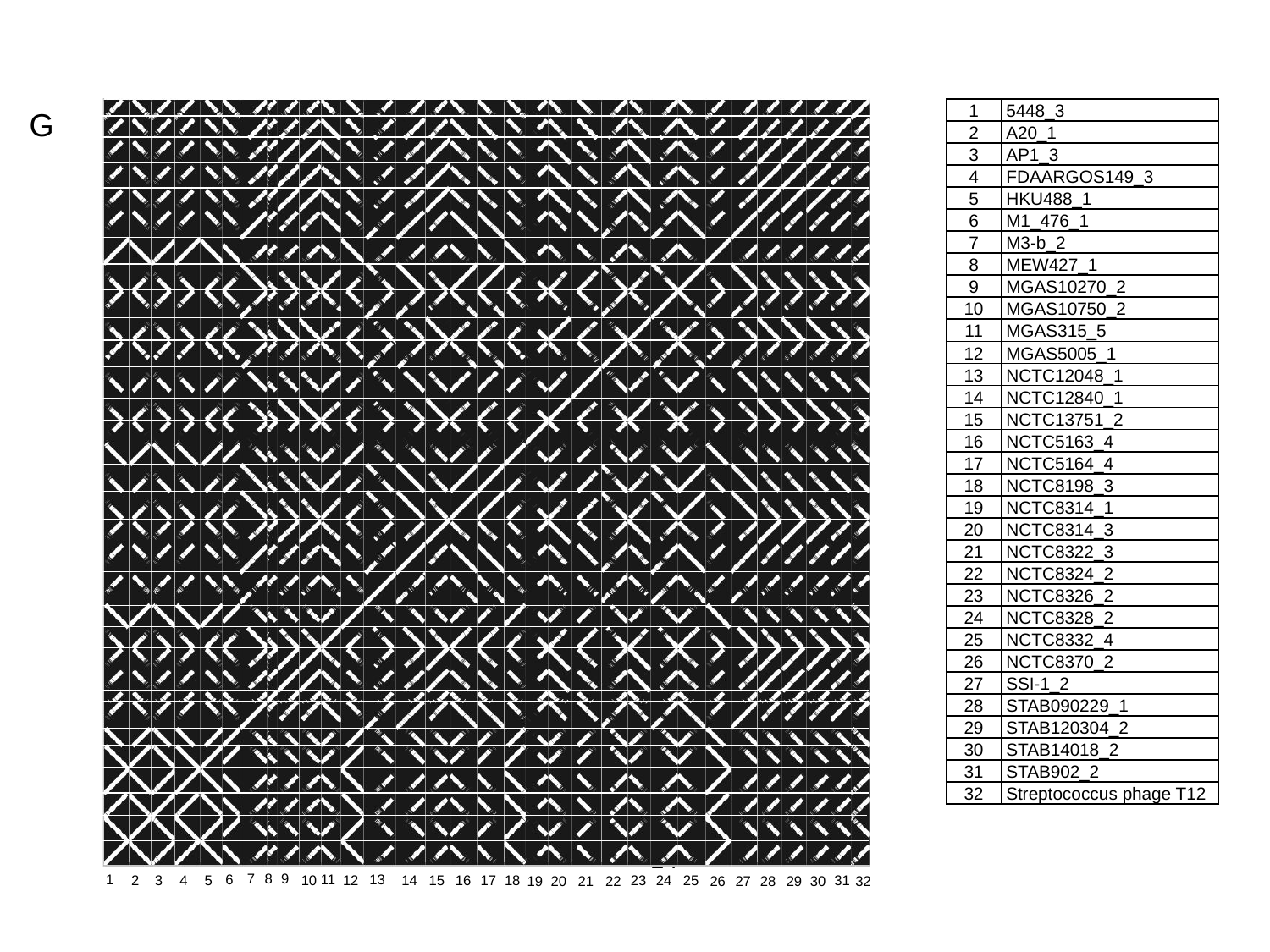

G
| 1 | 5448\_3 |
| --- | --- |
| 2 | A20\_1 |
| 3 | AP1\_3 |
| 4 | FDAARGOS149\_3 |
| 5 | HKU488\_1 |
| 6 | M1\_476\_1 |
| 7 | M3-b\_2 |
| 8 | MEW427\_1 |
| 9 | MGAS10270\_2 |
| 10 | MGAS10750\_2 |
| 11 | MGAS315\_5 |
| 12 | MGAS5005\_1 |
| 13 | NCTC12048\_1 |
| 14 | NCTC12840\_1 |
| 15 | NCTC13751\_2 |
| 16 | NCTC5163\_4 |
| 17 | NCTC5164\_4 |
| 18 | NCTC8198\_3 |
| 19 | NCTC8314\_1 |
| 20 | NCTC8314\_3 |
| 21 | NCTC8322\_3 |
| 22 | NCTC8324\_2 |
| 23 | NCTC8326\_2 |
| 24 | NCTC8328\_2 |
| 25 | NCTC8332\_4 |
| 26 | NCTC8370\_2 |
| 27 | SSI-1\_2 |
| 28 | STAB090229\_1 |
| 29 | STAB120304\_2 |
| 30 | STAB14018\_2 |
| 31 | STAB902\_2 |
| 32 | Streptococcus phage T12 |
2
3
13
16
17
18
4
5
1
6
7
8
9
10
11
12
15
14
8
7
9
6
1
13
11
12
3
4
5
18
10
24
16
2
25
14
17
31
23
15
29
32
21
30
19
22
28
20
26
27

## Slide 8
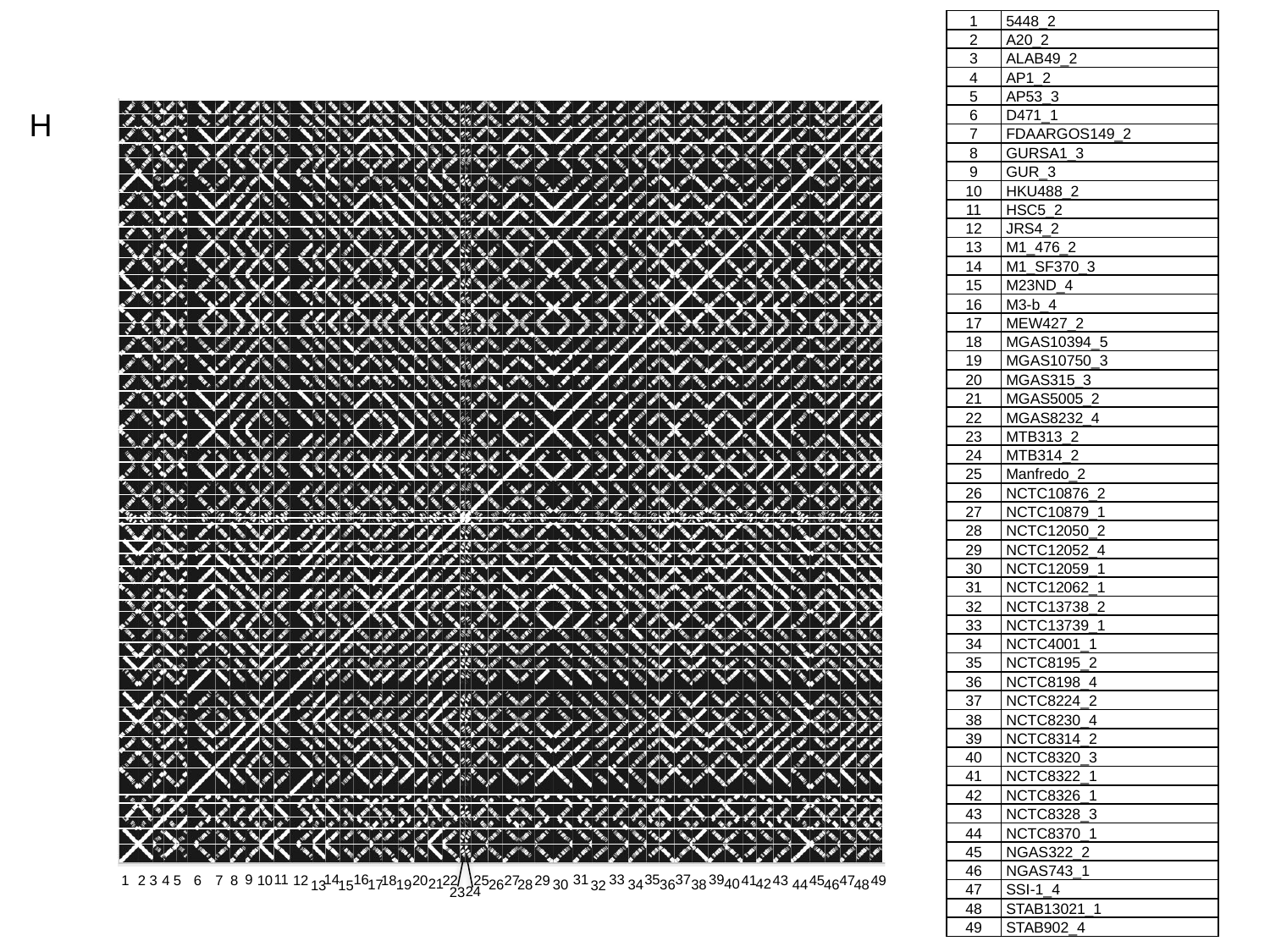

| 1 | 5448\_2 |
| --- | --- |
| 2 | A20\_2 |
| 3 | ALAB49\_2 |
| 4 | AP1\_2 |
| 5 | AP53\_3 |
| 6 | D471\_1 |
| 7 | FDAARGOS149\_2 |
| 8 | GURSA1\_3 |
| 9 | GUR\_3 |
| 10 | HKU488\_2 |
| 11 | HSC5\_2 |
| 12 | JRS4\_2 |
| 13 | M1\_476\_2 |
| 14 | M1\_SF370\_3 |
| 15 | M23ND\_4 |
| 16 | M3-b\_4 |
| 17 | MEW427\_2 |
| 18 | MGAS10394\_5 |
| 19 | MGAS10750\_3 |
| 20 | MGAS315\_3 |
| 21 | MGAS5005\_2 |
| 22 | MGAS8232\_4 |
| 23 | MTB313\_2 |
| 24 | MTB314\_2 |
| 25 | Manfredo\_2 |
| 26 | NCTC10876\_2 |
| 27 | NCTC10879\_1 |
| 28 | NCTC12050\_2 |
| 29 | NCTC12052\_4 |
| 30 | NCTC12059\_1 |
| 31 | NCTC12062\_1 |
| 32 | NCTC13738\_2 |
| 33 | NCTC13739\_1 |
| 34 | NCTC4001\_1 |
| 35 | NCTC8195\_2 |
| 36 | NCTC8198\_4 |
| 37 | NCTC8224\_2 |
| 38 | NCTC8230\_4 |
| 39 | NCTC8314\_2 |
| 40 | NCTC8320\_3 |
| 41 | NCTC8322\_1 |
| 42 | NCTC8326\_1 |
| 43 | NCTC8328\_3 |
| 44 | NCTC8370\_1 |
| 45 | NGAS322\_2 |
| 46 | NGAS743\_1 |
| 47 | SSI-1\_4 |
| 48 | STAB13021\_1 |
| 49 | STAB902\_4 |
H
37
35
39
16
31
33
11
9
14
12
3
29
6
4
5
20
1
18
43
45
10
41
49
2
8
27
7
25
22
47
40
42
21
19
17
26
48
36
28
38
46
44
34
30
15
32
13
24
23

## Slide 9
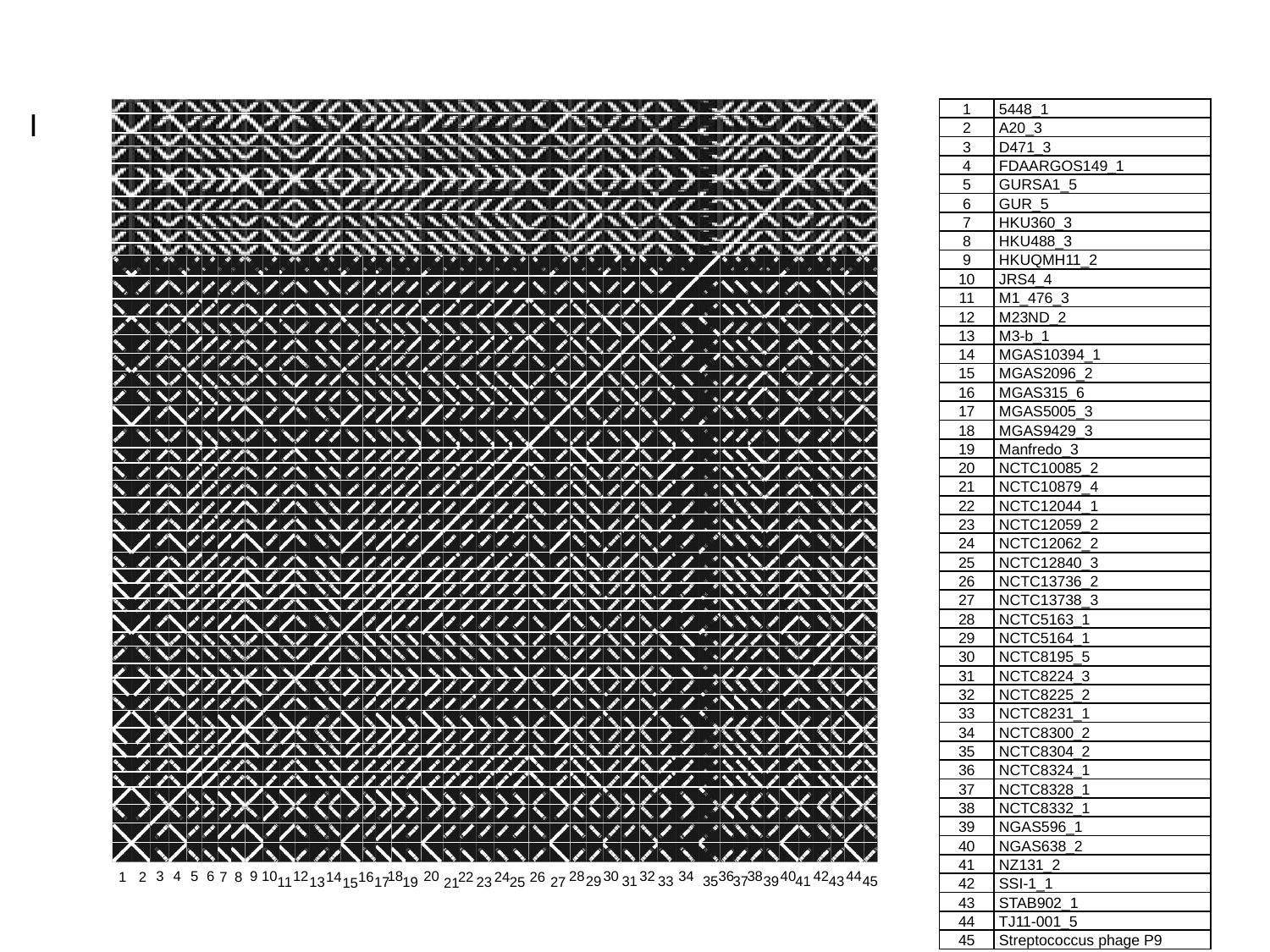

I
| 1 | 5448\_1 |
| --- | --- |
| 2 | A20\_3 |
| 3 | D471\_3 |
| 4 | FDAARGOS149\_1 |
| 5 | GURSA1\_5 |
| 6 | GUR\_5 |
| 7 | HKU360\_3 |
| 8 | HKU488\_3 |
| 9 | HKUQMH11\_2 |
| 10 | JRS4\_4 |
| 11 | M1\_476\_3 |
| 12 | M23ND\_2 |
| 13 | M3-b\_1 |
| 14 | MGAS10394\_1 |
| 15 | MGAS2096\_2 |
| 16 | MGAS315\_6 |
| 17 | MGAS5005\_3 |
| 18 | MGAS9429\_3 |
| 19 | Manfredo\_3 |
| 20 | NCTC10085\_2 |
| 21 | NCTC10879\_4 |
| 22 | NCTC12044\_1 |
| 23 | NCTC12059\_2 |
| 24 | NCTC12062\_2 |
| 25 | NCTC12840\_3 |
| 26 | NCTC13736\_2 |
| 27 | NCTC13738\_3 |
| 28 | NCTC5163\_1 |
| 29 | NCTC5164\_1 |
| 30 | NCTC8195\_5 |
| 31 | NCTC8224\_3 |
| 32 | NCTC8225\_2 |
| 33 | NCTC8231\_1 |
| 34 | NCTC8300\_2 |
| 35 | NCTC8304\_2 |
| 36 | NCTC8324\_1 |
| 37 | NCTC8328\_1 |
| 38 | NCTC8332\_1 |
| 39 | NGAS596\_1 |
| 40 | NGAS638\_2 |
| 41 | NZ131\_2 |
| 42 | SSI-1\_1 |
| 43 | STAB902\_1 |
| 44 | TJ11-001\_5 |
| 45 | Streptococcus phage P9 |
36
38
32
34
10
44
42
40
9
12
3
30
6
5
4
20
28
18
26
16
24
2
14
8
7
22
1
37
45
39
41
31
35
33
29
43
27
25
19
11
13
17
23
21
15

## Slide 10
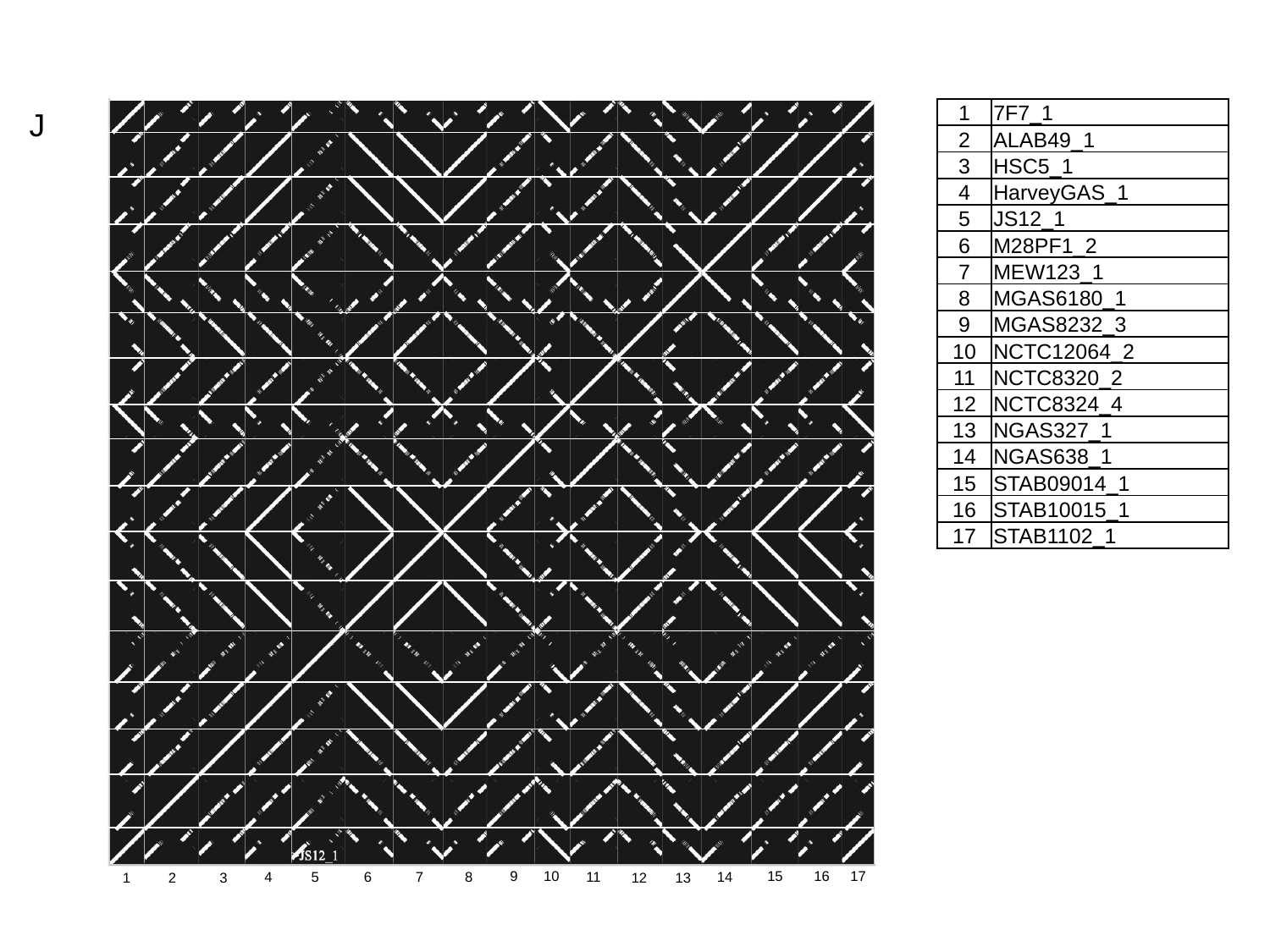

J
| 1 | 7F7\_1 |
| --- | --- |
| 2 | ALAB49\_1 |
| 3 | HSC5\_1 |
| 4 | HarveyGAS\_1 |
| 5 | JS12\_1 |
| 6 | M28PF1\_2 |
| 7 | MEW123\_1 |
| 8 | MGAS6180\_1 |
| 9 | MGAS8232\_3 |
| 10 | NCTC12064\_2 |
| 11 | NCTC8320\_2 |
| 12 | NCTC8324\_4 |
| 13 | NGAS327\_1 |
| 14 | NGAS638\_1 |
| 15 | STAB09014\_1 |
| 16 | STAB10015\_1 |
| 17 | STAB1102\_1 |
10
15
16
17
9
4
5
6
7
8
14
11
1
2
3
12
13

## Slide 11
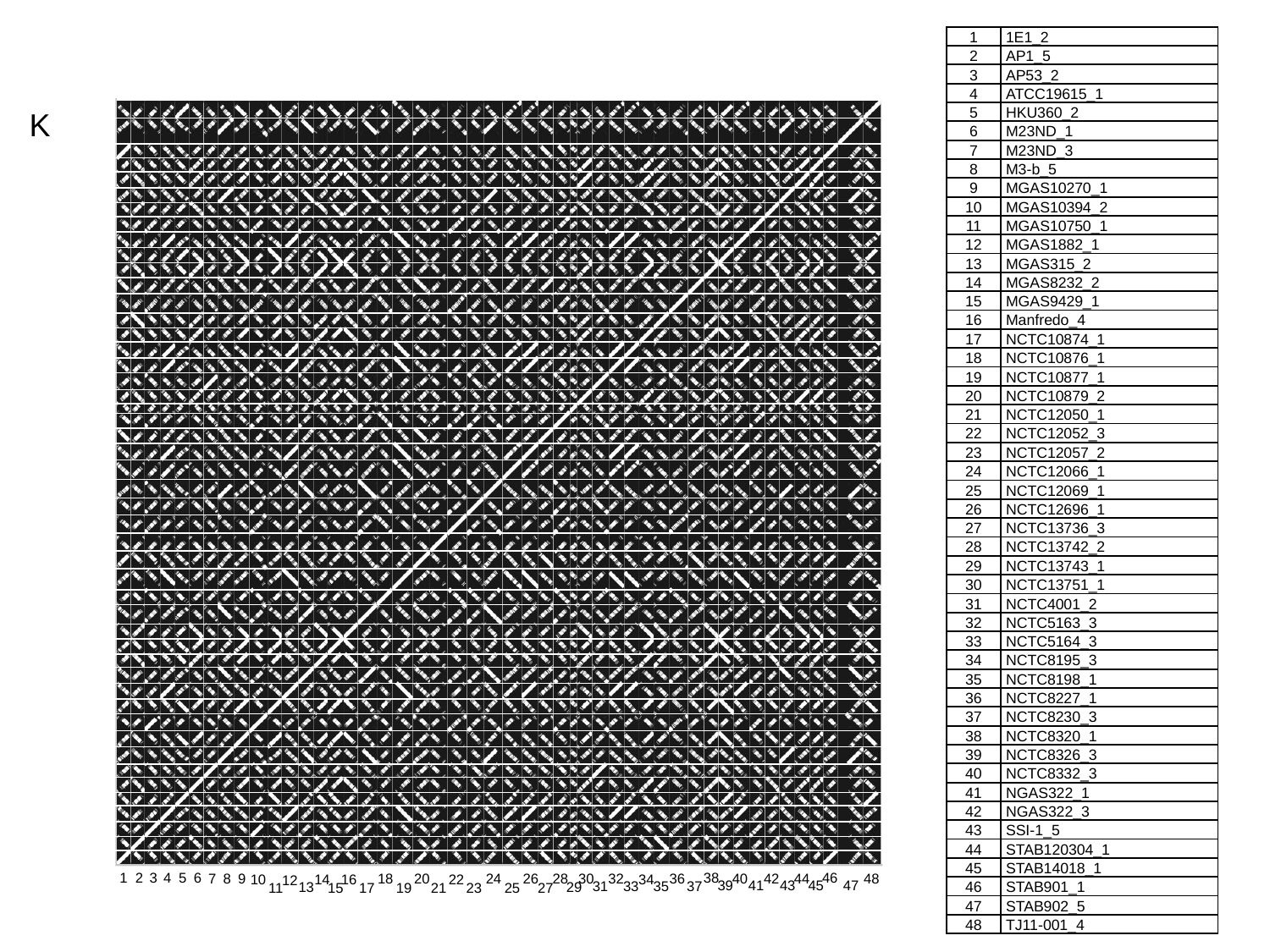

| 1 | 1E1\_2 |
| --- | --- |
| 2 | AP1\_5 |
| 3 | AP53\_2 |
| 4 | ATCC19615\_1 |
| 5 | HKU360\_2 |
| 6 | M23ND\_1 |
| 7 | M23ND\_3 |
| 8 | M3-b\_5 |
| 9 | MGAS10270\_1 |
| 10 | MGAS10394\_2 |
| 11 | MGAS10750\_1 |
| 12 | MGAS1882\_1 |
| 13 | MGAS315\_2 |
| 14 | MGAS8232\_2 |
| 15 | MGAS9429\_1 |
| 16 | Manfredo\_4 |
| 17 | NCTC10874\_1 |
| 18 | NCTC10876\_1 |
| 19 | NCTC10877\_1 |
| 20 | NCTC10879\_2 |
| 21 | NCTC12050\_1 |
| 22 | NCTC12052\_3 |
| 23 | NCTC12057\_2 |
| 24 | NCTC12066\_1 |
| 25 | NCTC12069\_1 |
| 26 | NCTC12696\_1 |
| 27 | NCTC13736\_3 |
| 28 | NCTC13742\_2 |
| 29 | NCTC13743\_1 |
| 30 | NCTC13751\_1 |
| 31 | NCTC4001\_2 |
| 32 | NCTC5163\_3 |
| 33 | NCTC5164\_3 |
| 34 | NCTC8195\_3 |
| 35 | NCTC8198\_1 |
| 36 | NCTC8227\_1 |
| 37 | NCTC8230\_3 |
| 38 | NCTC8320\_1 |
| 39 | NCTC8326\_3 |
| 40 | NCTC8332\_3 |
| 41 | NGAS322\_1 |
| 42 | NGAS322\_3 |
| 43 | SSI-1\_5 |
| 44 | STAB120304\_1 |
| 45 | STAB14018\_1 |
| 46 | STAB901\_1 |
| 47 | STAB902\_5 |
| 48 | TJ11-001\_4 |
K
2
38
46
3
6
4
5
1
44
8
7
40
42
30
9
32
26
48
36
28
20
24
18
10
34
22
16
14
12
43
45
41
47
39
37
35
31
33
13
29
27
25
21
23
11
19
15
17

## Slide 12
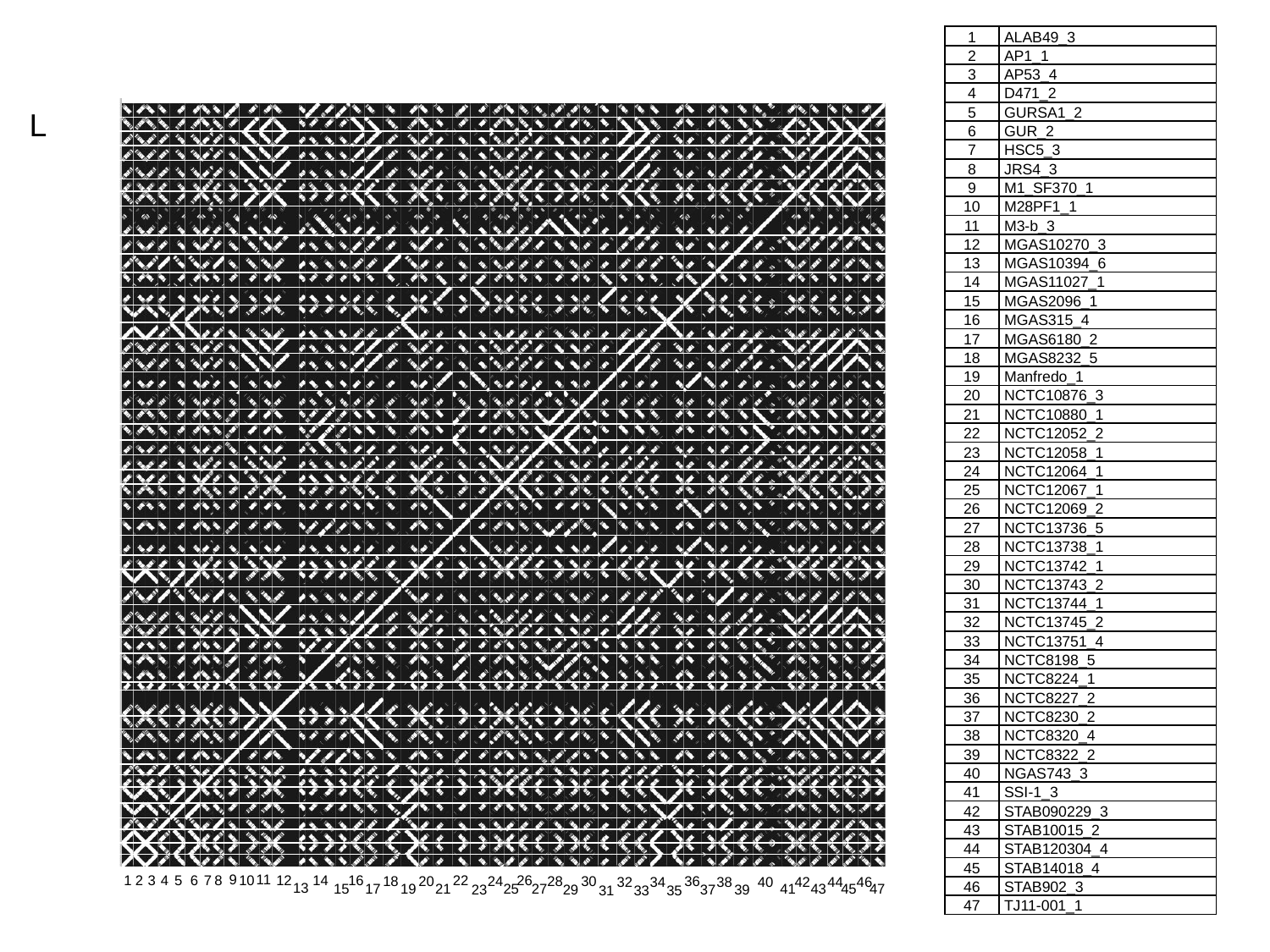

| 1 | ALAB49\_3 |
| --- | --- |
| 2 | AP1\_1 |
| 3 | AP53\_4 |
| 4 | D471\_2 |
| 5 | GURSA1\_2 |
| 6 | GUR\_2 |
| 7 | HSC5\_3 |
| 8 | JRS4\_3 |
| 9 | M1\_SF370\_1 |
| 10 | M28PF1\_1 |
| 11 | M3-b\_3 |
| 12 | MGAS10270\_3 |
| 13 | MGAS10394\_6 |
| 14 | MGAS11027\_1 |
| 15 | MGAS2096\_1 |
| 16 | MGAS315\_4 |
| 17 | MGAS6180\_2 |
| 18 | MGAS8232\_5 |
| 19 | Manfredo\_1 |
| 20 | NCTC10876\_3 |
| 21 | NCTC10880\_1 |
| 22 | NCTC12052\_2 |
| 23 | NCTC12058\_1 |
| 24 | NCTC12064\_1 |
| 25 | NCTC12067\_1 |
| 26 | NCTC12069\_2 |
| 27 | NCTC13736\_5 |
| 28 | NCTC13738\_1 |
| 29 | NCTC13742\_1 |
| 30 | NCTC13743\_2 |
| 31 | NCTC13744\_1 |
| 32 | NCTC13745\_2 |
| 33 | NCTC13751\_4 |
| 34 | NCTC8198\_5 |
| 35 | NCTC8224\_1 |
| 36 | NCTC8227\_2 |
| 37 | NCTC8230\_2 |
| 38 | NCTC8320\_4 |
| 39 | NCTC8322\_2 |
| 40 | NGAS743\_3 |
| 41 | SSI-1\_3 |
| 42 | STAB090229\_3 |
| 43 | STAB10015\_2 |
| 44 | STAB120304\_4 |
| 45 | STAB14018\_4 |
| 46 | STAB902\_3 |
| 47 | TJ11-001\_1 |
L
11
9
12
3
6
4
5
1
10
2
8
7
22
16
26
14
28
20
24
18
30
36
38
46
44
34
40
42
32
13
21
19
15
17
43
45
41
27
25
47
39
23
29
37
35
31
33

## Slide 13
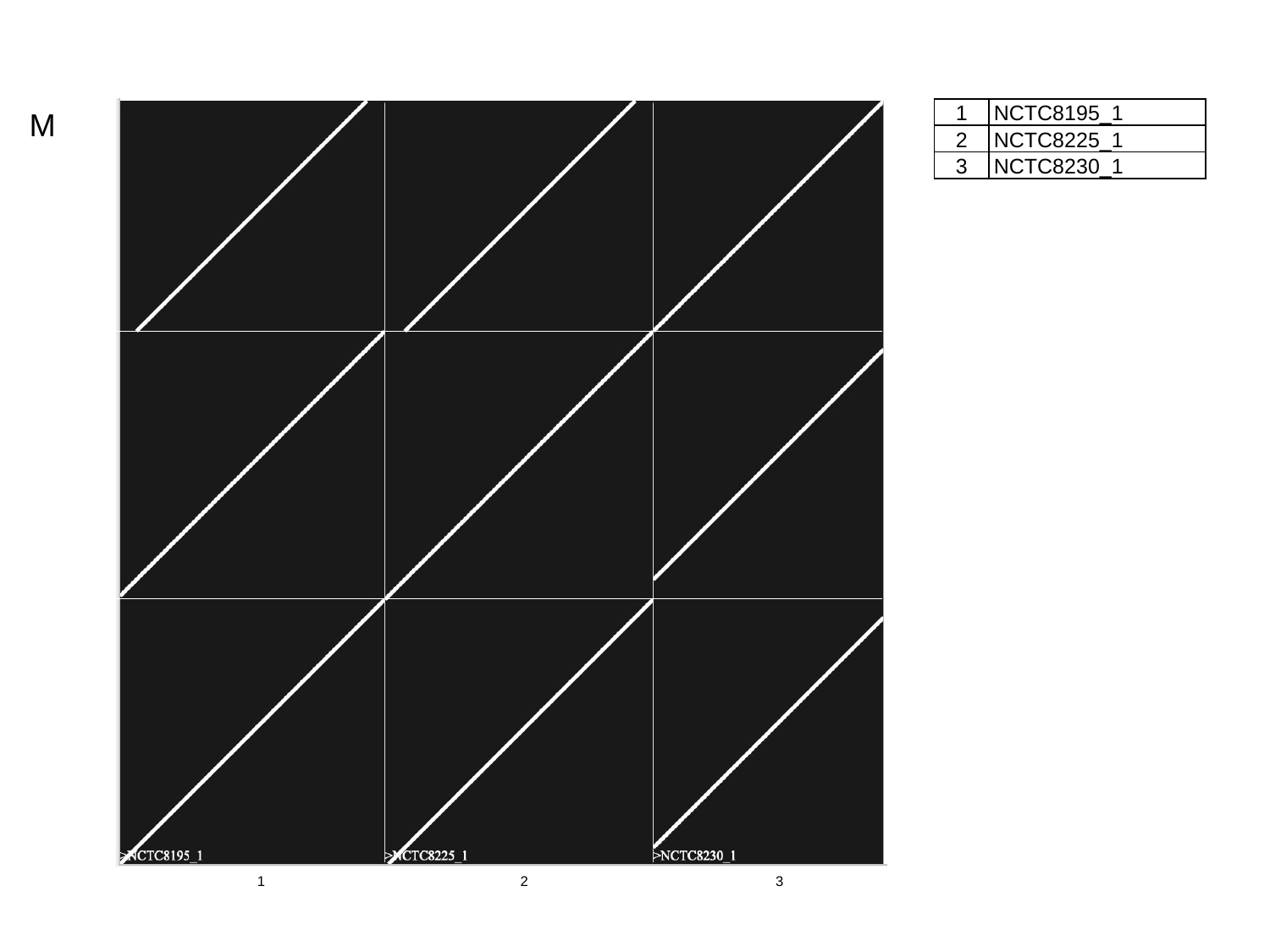

M
| 1 | NCTC8195\_1 |
| --- | --- |
| 2 | NCTC8225\_1 |
| 3 | NCTC8230\_1 |
1
2
3

## Slide 14
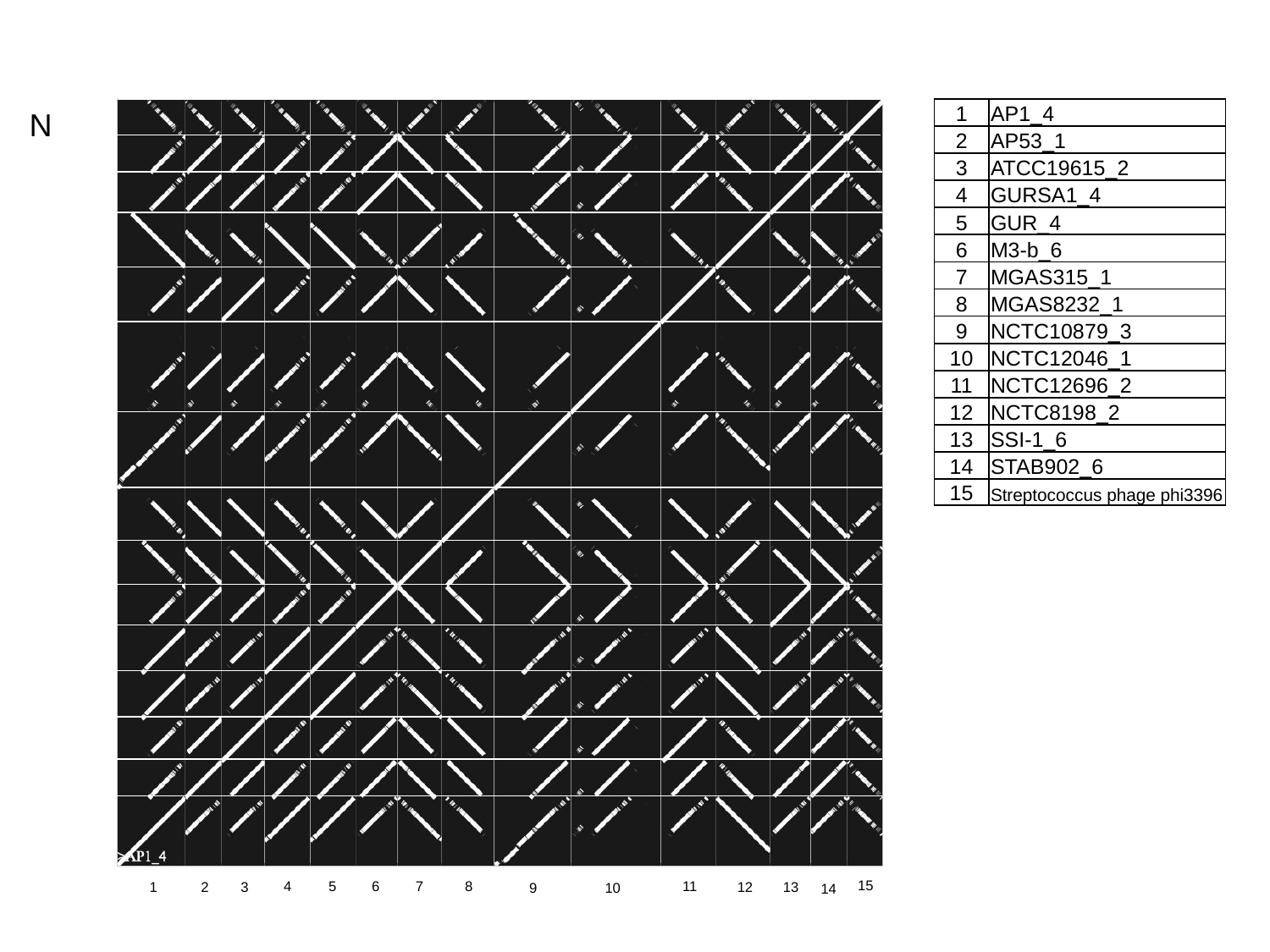

N
| 1 | AP1\_4 |
| --- | --- |
| 2 | AP53\_1 |
| 3 | ATCC19615\_2 |
| 4 | GURSA1\_4 |
| 5 | GUR\_4 |
| 6 | M3-b\_6 |
| 7 | MGAS315\_1 |
| 8 | MGAS8232\_1 |
| 9 | NCTC10879\_3 |
| 10 | NCTC12046\_1 |
| 11 | NCTC12696\_2 |
| 12 | NCTC8198\_2 |
| 13 | SSI-1\_6 |
| 14 | STAB902\_6 |
| 15 | Streptococcus phage phi3396 |
15
4
5
6
7
8
11
1
2
3
12
13
10
9
14

## Slide 15
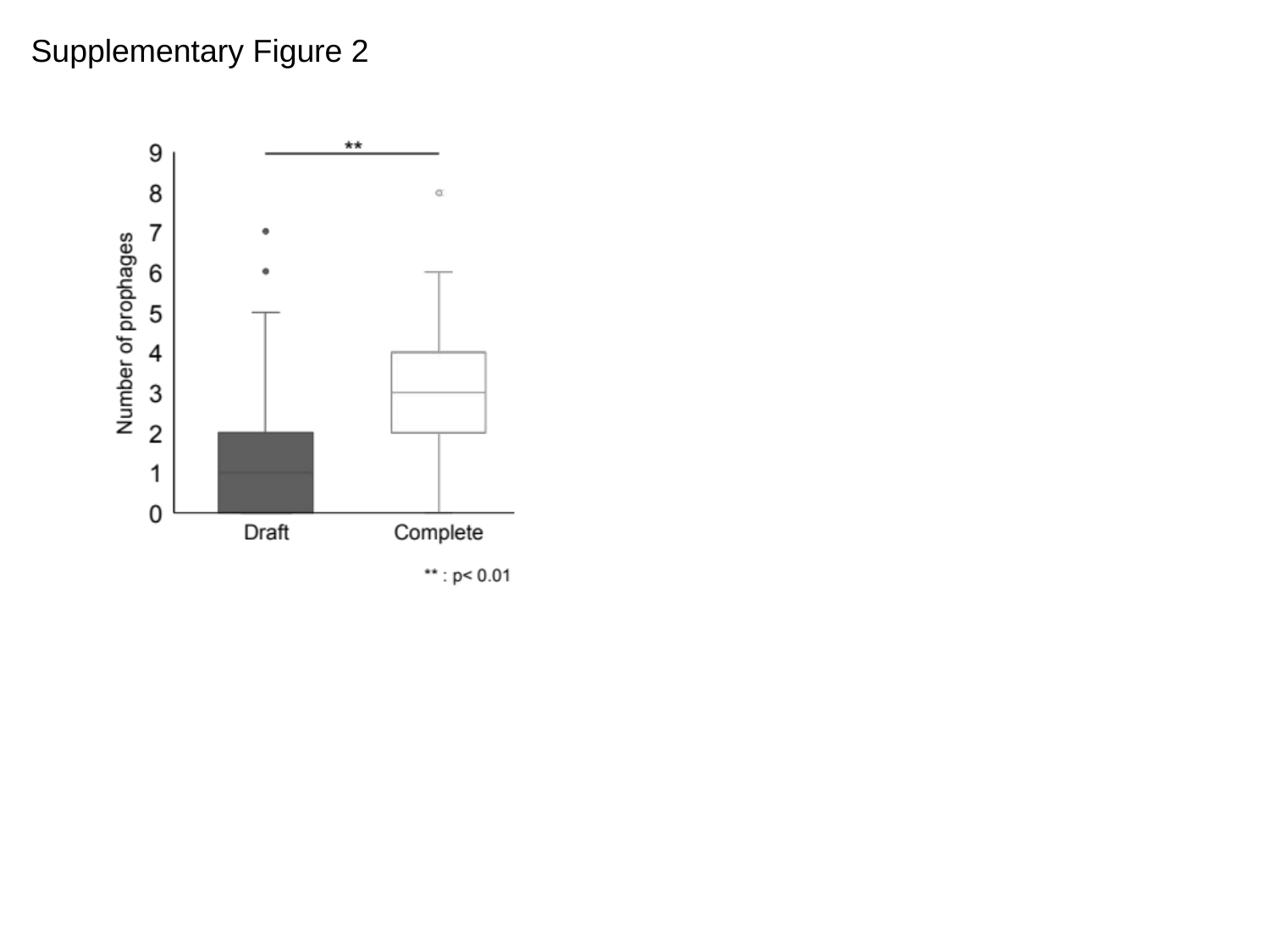

Supplementary Figure 2
